# Co-evolution of Bacterial Ribosomal Protein S15 with Diverse mRNA Regulatory Structures

**DOI:** 10.1371/journal.pgen.1005720

**Published:** 2015-12-16

**Authors:** Betty L. Slinger, Hunter Newman, Younghan Lee, Shermin Pei, Michelle M. Meyer

**Affiliations:** Department of Biology, Boston College, Chestnut Hill, Massachusetts, United States of America; Fred Hutchinson Cancer Research Center, UNITED STATES

## Abstract

RNA-protein interactions are critical in many biological processes, yet how such interactions affect the evolution of both partners is still unknown. RNA and protein structures are impacted very differently by mechanisms of genomic change. While most protein families are identifiable at the nucleotide level across large phylogenetic distances, RNA families display far less nucleotide similarity and are often only shared by closely related bacterial species. Ribosomal protein S15 has two RNA binding functions. First, it is a ribosomal protein responsible for organizing the rRNA during ribosome assembly. Second, in many bacterial species S15 also interacts with a structured portion of its own transcript to negatively regulate gene expression. While the first interaction is conserved in most bacteria, the second is not. Four distinct mRNA structures interact with S15 to enable regulation, each of which appears to be independently derived in different groups of bacteria. With the goal of understanding how protein-binding specificity may influence the evolution of such RNA regulatory structures, we examine whether examples of these mRNA structures are able to interact with, and regulate in response to, S15 homologs from organisms containing distinct mRNA structures. We find that despite their shared RNA binding function in the rRNA, S15 homologs have distinct RNA recognition profiles. We present a model to explain the specificity patterns observed, and support this model by with further mutagenesis. After analyzing the patterns of conservation for the S15 protein coding sequences, we also identified amino acid changes that alter the binding specificity of an S15 homolog. In this work we demonstrate that homologous RNA-binding proteins have different specificity profiles, and minor changes to amino acid sequences, or to RNA structural motifs, can have large impacts on RNA-protein recognition.

## Author Summary

RNA-protein interactions are important for many biological processes, and aberrant interactions have been implicated in many human diseases. However, how the specificity of such interactions may change over time and impact the evolution of both partners is still largely unexplored. In this work we examine four homologs of ribosomal protein S15 from diverse bacterial species. While S15 interacts with a conserved rRNA site, each S15 homolog regulates its own expression using a different mRNA structure. We measured the *in vitro* and regulatory interactions for all pairwise combinations between four S15 homologs and regulatory mRNA structures from diverse species. We find that each S15 homolog interacts with a different set of mRNA structures and their mutants. This work demonstrates that even highly conserved proteins such as S15 can have distinct RNA-binding repertoires that are likely the result of selection on both the mRNA and the protein sequence over time.

## Introduction

RNA-protein interactions and ribonucleoprotein complexes play key roles in many cellular processes including transcriptional regulation, translation, epigenetic regulation, and post-transcriptional silencing [1]. Furthermore, aberrant RNA-protein recognition and binding has been linked to multiple human disease states [1–4], as well as promoting cancer metastasis [5], and tumorigenesis [6–8]. Efforts to characterize RNA-protein interactions have focused on identification of RNA binding sites using experimental data coupled with motif-finders. These approaches typically identify short conserved sequences (*k*-mers) that may also have specific positioning within a predicted RNA secondary structure [9,10]. However, many RNA-protein interactions involve complex three-dimensional interactions that are not easily captured by *k*-mer descriptions[11]. Several studies have tried to identify general rules governing RNA-protein interactions using the growing collection of structural data [12,13]. Although limited by the availability of non-redundant data, these studies show that both electrostatic interactions and shape complementarity, on the part of both the RNA and the protein, are important for recognition.

Due to the complexity of RNA-protein interactions, and the challenges associated with in-depth characterization of RNA binding sites, relatively few studies have assessed how the specificities of RNA-binding proteins may be conserved, or altered over evolutionary time. Many eukaryotic RNA-binding proteins appear to have conserved recognition motifs [14]. However, there may be multiple modes of binding for a single protein (e.g. PUF (Pumilio and FBF) RNA-binding proteins), and minor changes to a protein sequence can have specific effects on RNA recognition [15]. Due to the nature of the genetic code, the direct impacts of genomic change on the structure of proteins and RNA are very different. RNA secondary structure is more conserved than sequence within RNA families [16]. Amino acid sequences of proteins tend to be much more highly conserved than nucleotide sequences of structured RNAs, and it is often difficult or impossible to follow the vertical inheritance of any but the most conserved structured RNAs (e.g. the ribosome) across large evolutionary distances [17].

For many RNA regulatory functions it appears that there are different RNA structures that accomplish very similar or identical biological functions in different bacterial phyla, which adds to the complexity of describing these RNA-protein interactions over evolutionary time. Bacteria commonly use portions of their mRNA transcripts as *cis*-acting regulatory elements (riboregulators). Such regulators typically alter RNA structure in response to cellular cues including small molecules (riboswitches), tRNAs (t-boxes), or proteins [18,19]. A classic example of the structural diversity that has arisen across different bacterial phyla are the many distinct riboswitch classes that bind the small molecule *S*-adenosyl methionine (SAM) [20–24]. From structural data it is clear that at least three of these RNAs interact with their ligand (SAM) in fundamentally different ways, suggesting completely independent derivation [25–28]. Furthermore, this example is far from unique. Two distinct riboswitch classes interact with the second messenger c-di-GMP [29,30], and three such classes with the nucleoside prequeosine-1 [31–33].

The existence of multiple unique RNA architectures responsible for analogous biological functions is not limited to RNA-small molecule interactions. This phenomenon is also apparent for riboregulators interacting with protein partners. Multiple mRNA regulatory structures have been identified that perform autogenous regulation in response to ribosomal proteins bL20, uS4, and uS15 [34–37]. From even this small set of RNA-protein interactions, we see that distinct RNA architectures in different bacterial phyla can successfully perform analogous biological functions by interacting with homologous protein binding partners. In some cases there is obvious similarity between the mRNA and rRNA binding-sites, suggesting that the protein recognizes the same tertiary structure features [38,39]. However, there are several examples where this similarity is not obvious [40–43]. In such cases, it remains unclear how much of the mRNA structural diversity observed is due to independent derivation of the similar tertiary structure, or if differences between homologous protein partners lead to distinct RNA-binding profiles.

**Fig 1:**
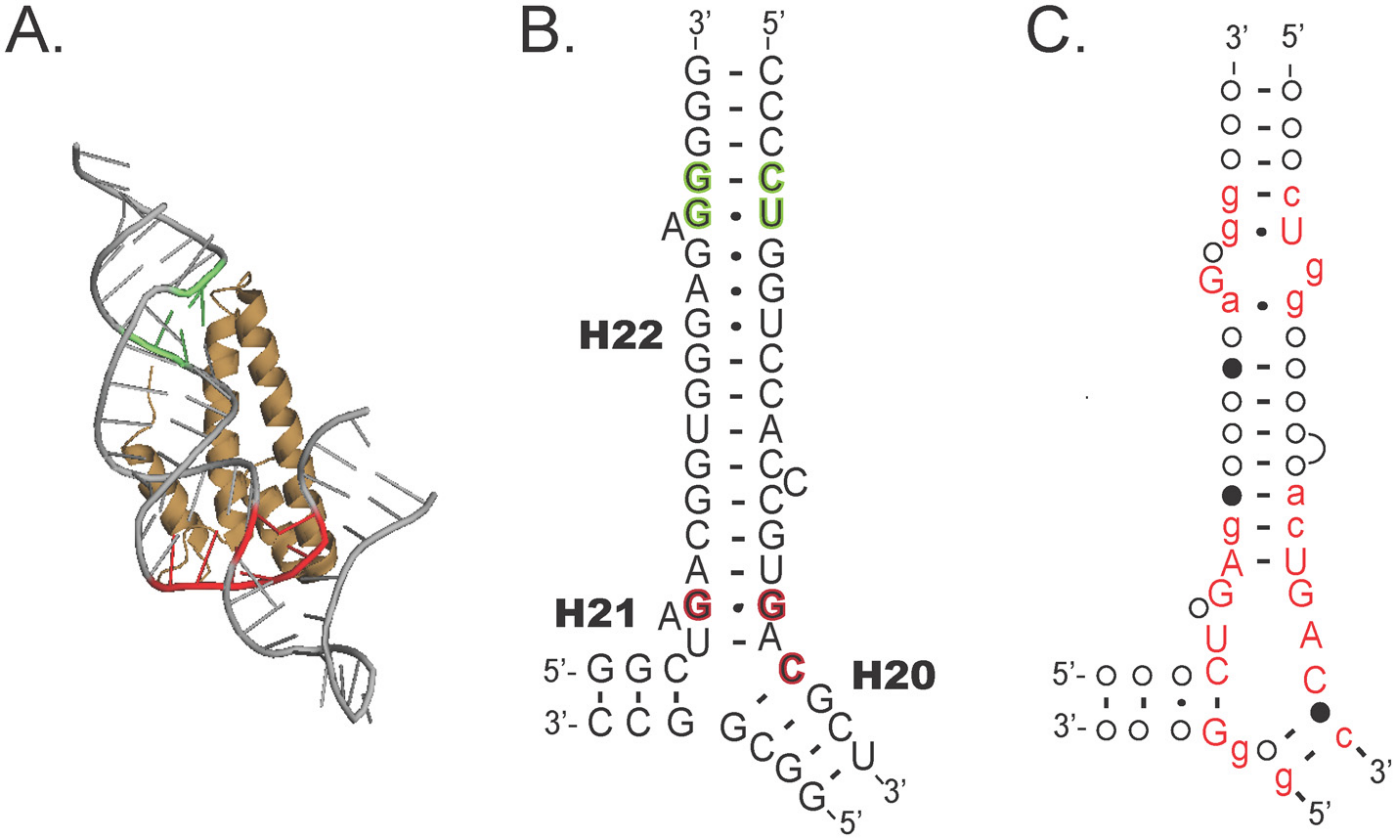
Overview of S15-rRNA binding (A) S15 (tan) binds ribosomal RNA (gray) at two independent sites. The first site is formed at the junction of three helices where a GGC base triple interacts with S15 (red). In this area, portions of S15 alpha helix 2 and 3 (S15-α2/3) contact the three-way junction. The second major site of interaction is at a GU/GC motif in helix 22 (green, H22). This RNA motif is recognized by residues in the S15 Loop 2 region. Figure was generated using PyMOL and crystal structure data from *T*. *thermophilus* [65]. *(B)* Secondary structure of the *E*. *coli* S15-binding region of the rRNA highlighting the GGC base triple (red), and the GU/GC motif (green). *(C)* Conservation of the rRNA across all bacteria (adapted from data at the Comparative RNA Website [45]), upper case red letters are conserved >98%, lower case letters 90–98%, closed circle 80–90%, and open circle <80% conserved.

To assess these questions, we have focused on ribosomal protein S15 which has two RNA-binding roles. S15 is a primary rRNA binding protein that is responsible for organizing the 16S rRNA during assembly of the small ribosomal subunit. The primary rRNA-S15 recognition site is formed where helices H20, H21, and H22 come together to yield a three-way junction (3WJ) that contains the base-triple GGC [44] (Fig 1A and 1B). The secondary S15-recognition site is a GU/GC motif that is ~10 nucleotide pairs (1 helical turn) distal to the 3WJ in H22 (Fig 1A and 1B). Together the 3WJ and the GU/GC motif form a bipartite S15-recognition surface in the rRNA. Not surprisingly, this region of the rRNA is highly conserved across all bacterial species to ensure proper ribosome assembly and function [45] (Fig 1C).

**Fig 2:**
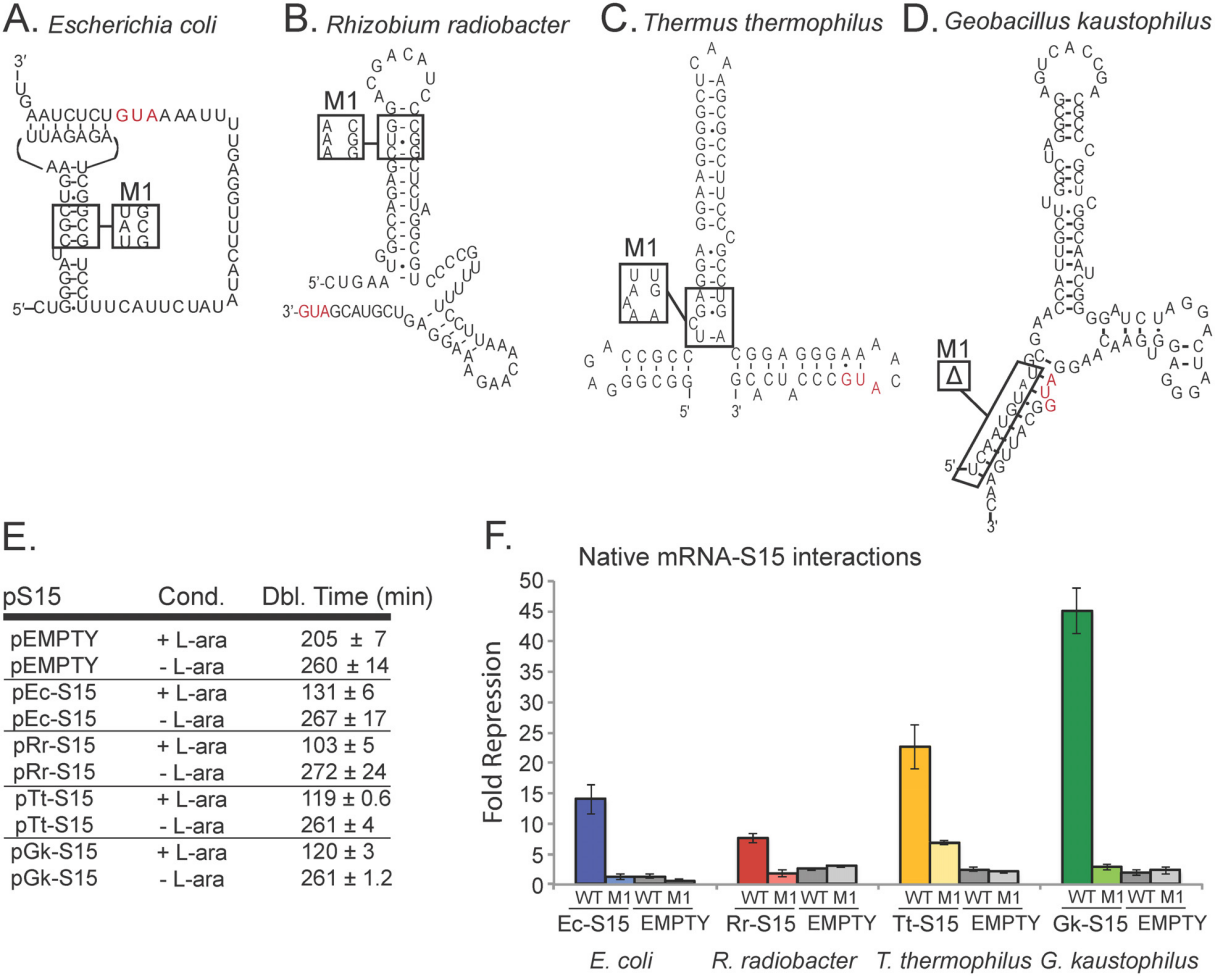
Native mRNA-S15 regulation is observed for mRNA structures that interact with S15 homologs in different bacterial species. *(A)* Ec-mRNA from *E*. *coli* and Ec-mRNA-M1; *(B)* Rr-mRNA from *R*. *radiobacter* and Rr-mRNA-M1*; (C)* Tt-mRNA from *T*. *thermophilus* and Tt-mRNA-M1; *(D)* Gk-mRNA from *G*. *kaustophilus* and Gk-mRNA-M1; *(E)* Doubling times calculated during logarithmic phase growth for Δ*rpsO* strain carrying plasmids that express different S15 homologs (pEc-S15, pRr-S15, pTt-S15, pGk-S15) or the vector with no protein insert (pEMPTY) under conditions where protein is expressed (+arabinose) and not expressed (-arabinose). (*F*) Fold-repression for each mRNA with its native binding partner. Fold-repression corresponds to (β-galactosidase activity (+arabinose))/(β-galactosidase activity (-arabinose)). β-galactosidase units under each condition are in S2 Fig. Each mRNA is compared to its own mutant (eg. Ec-mRNA and Ec-mRNA-M1 are compared in the same set of bars). Error bars represent standard error across 3 or more biological replicates.

S15 also interacts with an RNA structure found in the 5’-UTR of its own transcript enabling negative regulation of its own expression in many bacterial species. However, unlike the rRNA, this regulatory structure is not conserved [35]. In different groups of bacteria, four experimentally validated [37,44,46,47], as well as two predicted [37], mRNA structures with distinct architectures interact with their respective S15 homologs to enable gene regulation (Fig 2A–2D).

In this study we assess the interactions between examples of each of the S15-interacting mRNA families and their respective S15 homologs. These natural mRNA-protein interactions provide us with an opportunity to explore whether the diverse RNA architectures present similar tertiary structure surfaces to the protein, or if the different S15 protein homologs have distinct RNA recognition profiles. We assess RNA-S15 recognition using a translationally fused β-galactosidase reporter to characterize biologically relevant regulatory interactions, and use *in vitro* binding assays to directly quantify the RNA-protein interactions. We find that the results of the regulatory assays and *in vitro* assays largely agree and together show that there are differences between S15 homologs that result in specific recognition of the diverse mRNA structures. Furthermore, we analyze the conservation of S15 amino acid sequences from species showing different recognition patterns and identify amino acid mutations responsible for these specificity changes. Together our results suggest that even highly conserved RNA-binding proteins have distinct RNA recognition profiles, and that co-evolution has occurred between bacterial S15 homologs and their respective mRNA regulators.

## Results and Discussion

### Regulation assays confirm native mRNA-S15 interactions

To explore whether S15 homologs can specifically recognize different mRNA architectures to allow regulation within the cell, we utilized a β-galactosidase reporter assay. This functional assay directly tests the regulatory interaction between an mRNA and ribosomal protein S15 and enables the mRNA to fold into a biologically relevant structure. One plasmid contains an mRNA-LacZ fusion (pRNA) which was constructed by cloning the 5’-UTR through the first 5–9 codons of *rpsO* in-frame with *lacZ* and downstream of an IPTG-inducible promoter. A second plasmid (pS15) includes a full-length *rpsO* open reading frame (encoding S15) under the control of the pBAD33 L-arabinose inducible promoter. The plasmids have compatible replication origins and different antibiotic markers allowing them to be stably maintained in the same bacterium. For regulatory assays the plasmids are co-transformed into an *E*. *coli* K12:Δ*rpsO* strain that lacks endogenous S15 [48] (strain confirmed through PCR screening, S1 Fig). Each of the S15 homologs complemented this strain, enabling much faster growth when protein expression was induced (Fig 1E).

Cells containing a pRNA and a pS15 are grown with and without L-arabinose, and at stationary phase the reporter is induced for 30 minutes with the addition of IPTG. Subsequently, the β-galactosidase activity of + and–L-arabinose cultures started from a single colony are compared to indicate whether a given mRNA structure enables S15-dependent regulation of β-galactosidase expression. Given the short induction time during stationary phase, we did not observe any noticeable growth changes upon induction of individual mRNA reporter constructs.

The four experimentally validated riboregulators and their respective S15 homologs from *Escherichia coli* (Ec-mRNA, Ec-S15), *Geobacillus kaustophilus* (Gk-mRNA, Gk-S15), *Thermus thermophilus* (Tt-mRNA, Tt-S15), and *Rhizobium radiobacter* (Rr-mRNA, Rr-S15) were each examined using the β-galactosidase reporter assay. We confirmed all native mRNA-S15 regulatory interactions (Fig 2F) by directly comparing fold repression of pS15 to pBAD33 with no insert (pEMPTY). In each case we find that the native regulatory interaction can be detected using our assay in the surrogate organism. However, the unregulated levels of β-galactosidase expression using each mRNA riboregulator affects the resulting fold-repression (S2 Fig). The Ec-mRNA showed the highest β-galactosidase activity (~5,000–10,000 Miller Units) whereas the remaining mRNAs tested were all within a similar range (~1000–2000 Miller Units). To further ensure the significance of our observed interactions, a mutation abolishing the native binding interaction was introduced into each mRNA. In each case repression was reduced, typically to levels comparable to that observed for pEMPTY (~2-fold), although the Tt-mRNA-M1 does retain some regulatory activity (Fig 2F).

### Regulation assays reveal specific RNA recognition patterns

**Fig 3:**
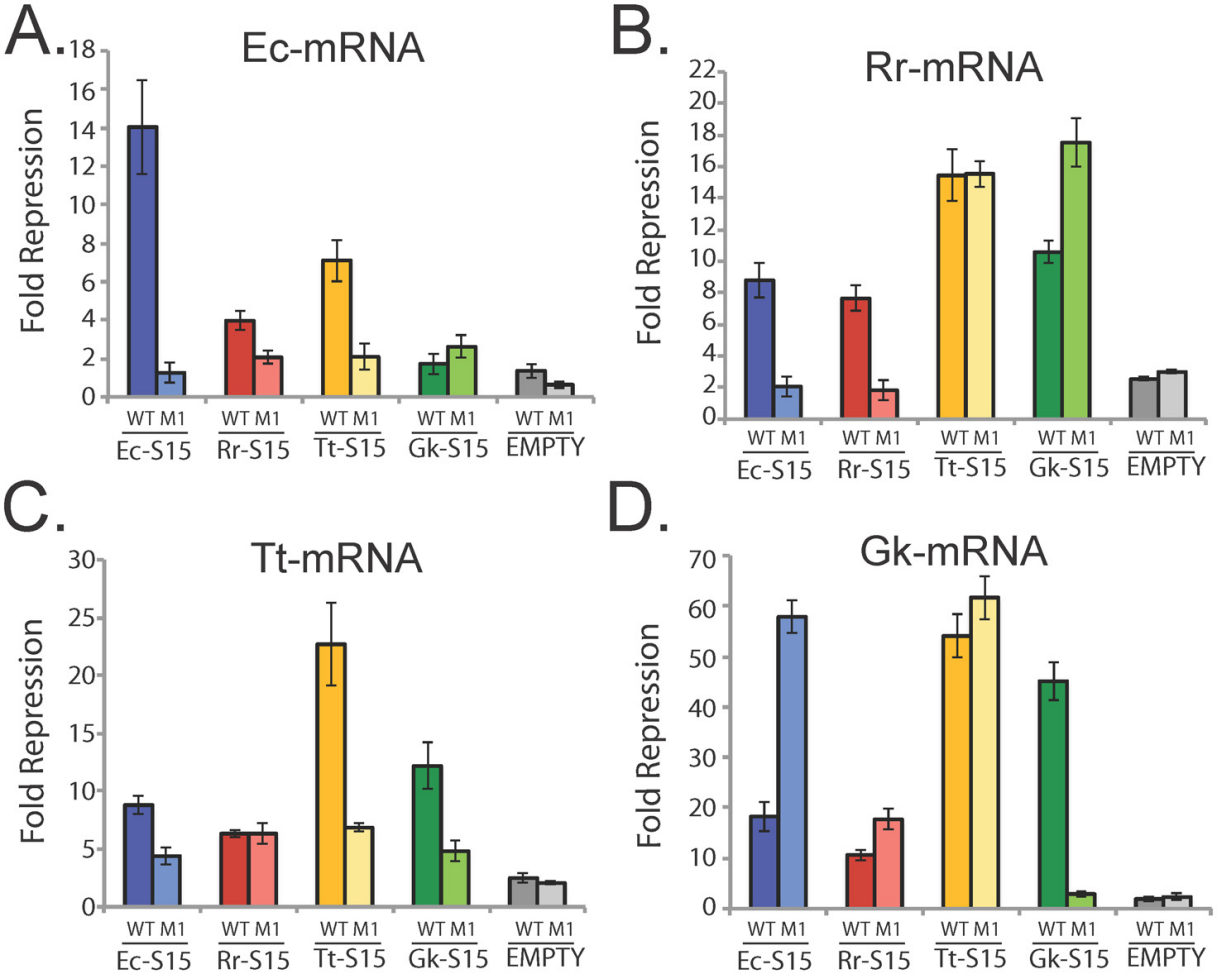
Inter-species regulatory activity (fold-repression) of each mRNA in response to each S15 homolog. *(A)* Ec-mRNA; *(B)* Rr-mRNA; *(C)* Tt-mRNA *(D)* Gk-mRNA. β-galactosidase units under each condition are in S2 Fig. Each mRNA is compared to its mutant and to pEMPTY (see S2 Table). Error bars correspond to standard error for 3 or more replicates. Data corresponding to native interactions is re-plotted from Fig 2 for comparison.

To determine whether the distinct mRNA architectures contain a shared tertiary structure or binding motifs, we examined all inter-species interactions using our regulatory assay. These results show that each mRNA structure has a specific set of S15 homologs to which it responds. For the mRNA regulator from *E*. *coli*, Ec-mRNA, both Rr-S15 and Tt-S15 successfully regulated β-galactosidase expression, yet do so more modestly than its native binding partner, Ec-S15 (Fig 3A). The mutation abolishing the native RNA-protein interaction (Ec-mRNA-M1, derived from [49]) deregulated reporter expression in response to both Rr-S15 and Tt-S15. Gk-S15 did not regulate the Ec-mRNA or its mutant. These results suggest that these three S15 homologs, Ec-S15, Tt-S15, and Rr-S15, interact with this mRNA in a similar fashion to regulate gene expression. The inability of this mRNA to respond to Gk-S15 suggests that Gk-S15 requires a regulatory motif or structure not found in Ec-mRNA.

In contrast, the mRNA from *R*. *radiobacter*, Rr-mRNA, regulates gene expression in response to all the S15 homologs (Fig 3B). A mutation to Rr-mRNA in the main stem was sufficient to deregulate expression in response to both Ec-S15 and Rr-S15. However, this mutation did not impact the convincing regulation observed in response to Tt-S15 and Gk-S15 (>10-fold repression observed). This suggests that the Ec-S15 and Rr-S15 homologs utilize similar determinants to recognize the mRNA, but that the Gk-S15 and Tt-15 homologs may be recognizing alternative motifs that are not impacted by the mutation.

The mRNA from *T*. *thermophilus*, Tt-mRNA, displayed regulatory activity in response to all the S15 homologs. A mutation to the 3WJ (derived from [50]) diminishes Tt-mRNA’s response to Tt-S15, Ec-S15, and Gk-S15 homologs (Fig 3C). However, this mutation does not completely abolish regulation in response to any of the proteins, and had no effect on regulation in response to Rr-S15 (Fig 3C). These results have two potential interpretations. First, Tt-S15, Ec-S15, and Gk-S15 proteins may recognize Tt-mRNA in a different manner than Rr-S15, and therefore a mutation to the binding site for Tt-S15 may not impact binding and regulation in response to Rr-S15. A second explanation is that the relatively modest 6-fold regulation observed for Rr-S15 is an artifact of our regulatory assay.

The mRNA from *G*. *kaustophilus*, Gk-mRNA, is also responsive to all S15 homologs tested (Fig 3D). Like the Rr-RNA, the convincing regulatory responses to Ec-S15, Rr-S15, and Tt-S15 were not diminished by the mutation to Gk-mRNA (a truncation used during *in vitro* studies in [40] expected to disrupt the 3WJ), while regulation in response to Gk-S15 was abolished by this mutation. Like the Rr-mRNA, this data suggests that that the binding determinants for Tt-S15, Ec-S15, and Rr-S15 on Gk-mRNA are different from those of Gk-S15, and that different S15 homologs utilize distinct features to recognize the same mRNA.

Together, the regulatory assays show that there is extensive, but not universal cross-reactivity in the inter-species mRNA-S15 regulatory interactions. However, results obtained with mRNA mutants suggest that even mRNAs recognized by multiple S15 homologs are recognized using different determinants. In particular, for both the Gk-mRNA and the Rr-mRNA, mutations that abolish native interactions have little or no impact on interactions with other S15 homologs.

### *In vitro* binding assays also show distinct recognition profiles for S15 homologs

Given that many of our mutations that abolish native interactions still allowed regulation in response to other protein homologs, we used *in vitro* nitrocellulose filter-binding assays to directly measure the strength of RNA-protein binding interactions to corroborate our findings. All four S15 homologs were purified and nitrocellulose filter binding assays were performed for all cross-species interactions. We find that the dissociation constants for native interactions are in the 2–20 nM range. However, the native interactions were not always the strongest interactions. For example, Gk-S15 bound Tt-mRNA with an affinity that was almost an order of magnitude smaller than Tt-S15 (0.35 nM vs. 2.11 nM).

We were unsuccessful in demonstrating Ec-mRNA interactions with any S15 homolog including its native binding partner; therefore it was omitted from further study. The native Ec-mRNA interaction with Ec-S15 has been characterized *in vitro* in the past (K_D_ = 231 nM)[51]. Notably, this value is significantly higher than those that we measured for the other native interactions. Although a 3’-terminal [^32^P]pCp has been previously shown to decrease the K_D_ four-fold in truncated versions of this RNA [51], we found that labeling the full-length mRNA with [^32^P]pCp did not change our result. We did not explicitly test the truncated RNA since we are primarily interested in the wild-type interaction.

Aside from our inability to measure interactions with Ec-mRNA, we find that our *in vitro* findings closely follow the results of the regulatory assays. The Rr-mRNA was able to interact with all S15 homologs *in vitro*, and all are relatively strong interactions with dissociation constants ranging from 1 to ~30 nM (Table 1). The inactivating mutation (Rr-mRNA-M1) abolished interaction with Ec-S15, but had little impact on interactions with the Gk-S15 or Tt-S15 homologs. These data corroborate our results from the regulatory assay indicating that Gk-S15 and Tt-S15 interact with the Rr-mRNA-M1, and further indicates that Ec-S15, Gk-S15, and Tt-S15 homologs use distinct features to recognize this mRNA.

**Table 1:** Binding constant for *in vitro* nitrocellulose binding assays reported ± the standard deviation. Binding curves supporting these values in S3 Fig.

Protein mRNA	Ec-S15 K_D_ (nM)	Rr-S15 K_D_ (nM)	Tt-S15 K_D_ (nM)	Gk-S15 K_D_ (nM)
Ec-Wt	231*	>1000	>500	>400
Ec-M1	n/a	n/a	n/a	n/a
Rr-Wt	28.6 ± 4.8	14.5 ± 6.1	12.0 ± 7.0	1.23 ± 0.25
Rr-M1	>300	n/a	8.8 ± 1.8	11.8 ± 6.9
Tt-Wt	>500	>500	2.11 ± 0.25	0.35 ± 0.23
Tt-M1	>500	>500	>500	† 76.3 ± 5.7
Gk-Wt	112 ± 38	205 ± 142	0.62 ± 0.07	3.47 ± 6.0
Gk-M1	57.5 ± 19.7	>250	0.12 ± 0.03	>2000

^†^Maximum fraction bound < 20%.

*value from [51].

Tt-mRNA binds strongly to both Tt-S15 and Gk-S15, which corroborates our *in vivo* regulation findings. Conversely, Ec-S15 and Rr-S15 both do not bind Tt-mRNA *in vitro*, which makes interpreting the regulatory assay results less clear. They both displayed modest regulatory activity *in vivo*. Mutating Tt-mRNA decreased the regulatory response to Ec-S15, yet did not significantly impact the response to Rr-S15 (Fig 3C). However, neither Ec-S15 or Rr-S15 were able to bind this mutant *in vitro*. In comparison to Gk-S15 and Tt-S15, the dissociation constants measured for Ec-S15 and Rr-S15 tend to be significantly higher for all measured S15-mRNA interactions, indicating that perhaps these proteins behave less well *in vitro*. Alternatively, relatively high levels of noise in our regulatory assay (even empty vector controls typically display 2–3 fold repression) may bias our findings. Therefore, Ec-S15 may be able to regulate gene expression using Tt-mRNA structure because the regulatory activity decreased with the mutated mRNA. However, whether Rr-S15 interacts with the Tt-mRNA to allow regulation remains unclear. In addition, although regulation of the Tt-mRNA by Gk-S15 is significantly reduced by the Tt-mRNA-M1 mutation, Tt-mRNA-M1 is not sufficient to completely abolish *in vitro* binding of Gk-S15. However, the measured K_D_ is over two-orders of weaker (0.35 nM vs 76.3 nM), and the maximum fraction of RNA bound by the protein is <20% (S3 Fig), indicating that the *in vitro* interaction may be non-specific.

Gk-mRNA interacted with all four S15 homologs *in vitro* (Table 1). The strongest interaction was with Tt-S15, roughly an order of magnitude stronger than the native Gk-S15 interaction, and roughly three orders of magnitude stronger than with Ec-S15 and Rr-S15. In addition, the Ec-S15 and Tt-S15 homologs retain strong interactions with the Gk-mRNA-M1. This suggests that the retained regulation for this mutant in response to Ec-S15 and Tt-S15 is because these homologs still bind the mutant mRNA. In addition, while we do not measure any interaction between Rr-S15 and the Gk-mRNA-M1 (up to 250 nM Rr-S15), the interaction between Rr-S15 and Gk-mRNA is relatively weak in comparison to the other S15 homologs (K_D_ ~200 nM). Therefore, Rr-S15 may bind Gk-mRNA-M1 weakly, yet this interaction is sufficient to regulate reporter expression within cells. In conclusion, our *in vitro* results with the Gk-mRNA suggest that the regulatory interactions we observed between Ec-S15, Rr-S15, Tt-S15 and the Gk-mRNA and its mutant (Gk-mRNA-M1) are indeed due to differences in the way that the proteins interact with the mRNA.

In summary, we find that measuring cross-species interactions between S15 homologs and diverse mRNA structures using both regulatory assays and *in vitro* binding assays shows that the two approaches largely agree. While in isolation each type of assay is prone to various artifacts ranging from poor *in vitro* binding properties, to likely differences in protein expression levels in the surrogate organism, the large extent of agreement between our two assays significantly strengthens our conclusions. Overall, we find that Tt-S15 and Gk-S15 bind very tightly *in vitro*. This may be due to many factors including that the Tt-S15 and Gk-S15 homologs are both from thermophiles and may be more stable resulting in better *in vitro* binding characteristics. We also assessed Tt- and Gk-S15 *in vitro* binding at 55°C and found that no significant differences were detected at the higher temperature.

### S15 homologs recognize mRNAs via distinct motifs

**Fig 4:**
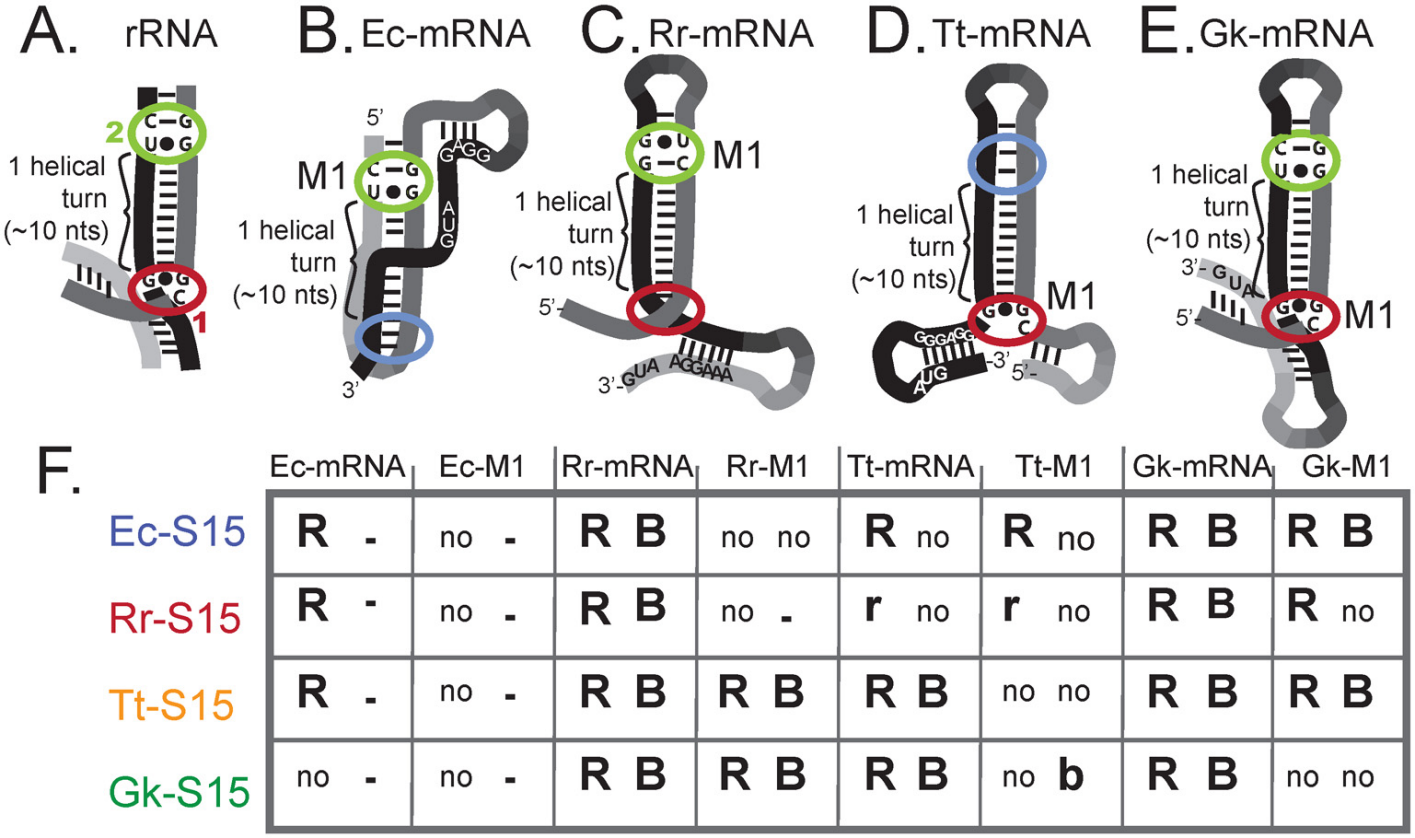
Summary of S15-mRNA binding sites and cartoon representation of RNA binding sites for S15. *(A)* rRNA; *(B)* Ec-mRNA; *(C)* Rr-mRNA; *(D)* Tt-mRNA; *(E)* Gk-mRNA. Regions circled in green putatively correspond to rRNA GU/GC motif, regions circled in red putatively correspond to three-way junction. Important aspects of the binding site as well as regulatory features such as Shine-Dalgarno sequences, start codons, and the regions targeted by mutations are indicated. *(F)* Table summarizing results from both regulatory assays and *in vitro* binding assays. “R” indicates regulatory activity observed, “r” indicates ambiguous regulatory activity observed, “B” indicates *in vitro* binding observed, “b” indicates marginal binding (<20% maximum fraction bound), “no” indicates no regulatory activity or no *in vitro* binding, and “–” indicates unmeasured.

To combine our *in vitro* and regulatory results into a single determination of whether or not an interaction occurs, we consider all measureable dissociation constants as viable interactions. For regulatory interactions, we consider all interactions that are significantly reduced by a mutation to the RNA, or corroborated by *in vitro* data as viable interactions (Fig 4F). Using this criterion there is only a single ambiguous interaction, which is that between Rr-S15 and the Tt-mRNA. In addition, we did not detect an *in vitro* interaction between Ec-S15 and the Tt-mRNA, although regulation was observed for this pairing (~ 10 fold repression), and it is reduced by the Tt-mRNA-M1, suggesting that it is not an artifact. From our collected data it is clear that there is extensive cross-reactivity, but that S15 homologs often recognize mRNAs using different characteristics, as demonstrated by the very divergent responses of different S15 homologs to the mutated mRNA structures. Using this data we can start to assess what RNA structural motifs result in these differences.

The rRNA binding site for S15 is bipartite, consisting of a three-way junction (3WJ) and a GU/GC motif approximately one helical turn away from the 3WJ (Fig 4A). Previous studies have established that the *E*. *coli* mRNA mimics of the GU/GC motif [51], and that the Tt-mRNA mimics the G-G-C base-triple found in the 3WJ of the rRNA [50]. However, in both of these cases it is clear that while the mRNA is contacted at a second position consistent with bi-partite binding, the second position bears limited resemblance to the rRNA. In the case of Ec-mRNA, the second binding site occurs within the co-axially stacked pseudoknot [51] (Fig 4B), and in the case of the Tt-mRNA, the long H2 stem is necessary for binding, but the GU/GC motif is replaced by a G•G mismatch [50] (Fig 4D). In contrast, the Gk-mRNA appears to contain mimics of both binding determinants. The 3WJ is mimicked in the multi-stem junction and a GU/GC motif is apparent approximately one helical turn away from this junction [46] (Fig 4E). In the case of the Rr-mRNA, far less data exists concerning which bases are necessary for binding. However, a GU/GC motif is apparent in the most conserved portion of the Rr-mRNA [37], and like the Tt-mRNA, the junction of the stems is important for retaining interaction with its native binding partner [37] (Fig 4C).

Taking our results in conjunction with previously published results, the data suggests that each of the mRNA structures mimics a portion of the rRNA. Both Ec-mRNA and Tt-mRNA contain a direct mimic for a portion of the binding site, while Gk- and Rr- mRNAs likely contain both portions (Fig 4). The inactivating mutations for each of the mRNAs target different portions of these rRNA binding sites. Rr-mRNA-M1 and Ec-mRNA-M1 both target putative GU/GC motifs, the Gk-mRNA-M1 is a truncation that presumably disrupts the 3-way junction, and the Tt-mRNA-M1 also targets the 3-way junction. The partial mimicry of the S15 rRNA binding site potentially explains the regulatory differences we observe for the S15 homologs.

Our observations suggest that Ec-S15 and Rr-S15 preferentially recognize the GU/GC motif. Regulatory interactions between both Ec-S15 and Rr-S15 and several mRNAs are significantly impacted when this region is mutated (Ec-mRNA-M1, and Rr-mRNA-M1). The regulatory interaction between Ec-S15 and Rr-S15 does not appear to be impacted by Gk-mRNA-M1, a mutant targeting the putative 3WJ. Tt-mRNA lacks the GU/GC motif, and while Ec-S15 appears to regulate gene expression using Tt-mRNA, this interaction could not be reproduced *in vitro*.

In contrast, the Gk-S15 appears to preferentially interact with a mimic of the three-dimensional motif formed at the helical junction. Gk-S15 does not interact with the Ec-mRNA (which lacks a mimic of the junction), it is not impacted by the Rr-mRNA-M1 mutation that targets the GU/GC motif, and mutations that impact the junction result in lack of regulatory activity (Tt-mRNA-M1, and Gk-mRNA-M1).

Finally, the Tt-S15 appears to regulate gene expression with any mRNA structure that contains either portion of the rRNA binding site. The Ec-mRNA and Tt-mRNA each contain an obvious mimic for a single portion of the rRNA binding site, and mutations to these regions prevent gene regulation in response to Tt-S15. The Gk-mRNA and Rr-mRNA are presumed to contain mimics of the entire rRNA binding site, and mutations that impact only one of these regions do not affect the regulatory interaction with Tt-S15. In summary, we propose that the four S15 homologs preferentially recognize different sections of the naturally occurring mRNA regulators.

**Fig 5:**
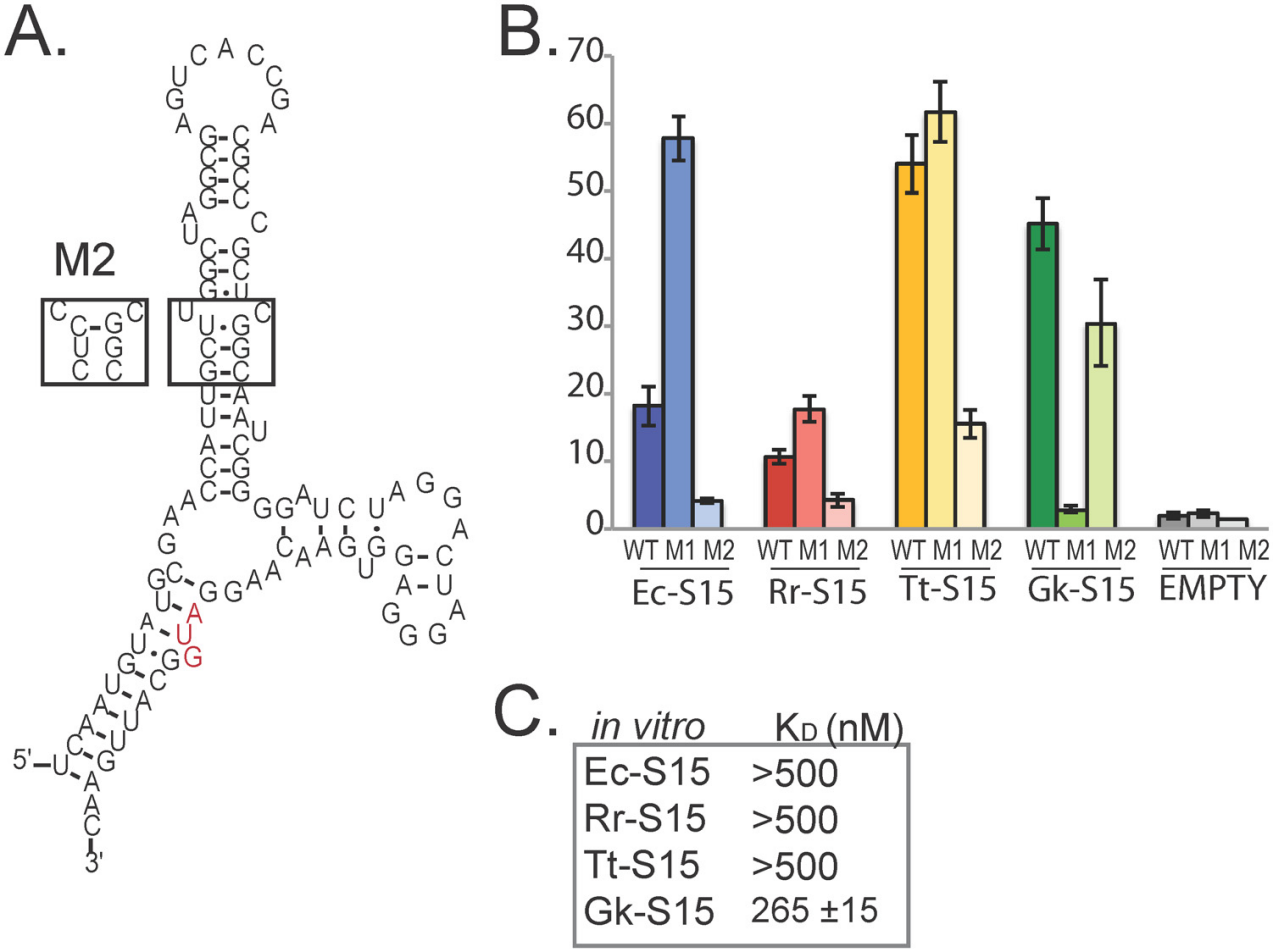
Second mutation to Gk-mRNA (Gk-mRNA-M2) strengthens model for interaction. *(A)* Mutation Gk-mRNA-M2 disrupts putative GU/GC motif. *(B)* Fold-repression for Gk-mRNA (WT), Gk-mRNA-M1, and Gk-mRNA-M2 in response to each S15 homolog and the empty vector (pEMPTY). β-galactosidase units under each condition are in S2 Fig. Data for Gk-mRNA and Gk-mRNA-M1 are re-plotted from Fig 3 for comparison. Error bars correspond to standard error for 3 or more replicates. (C) *In vitro* binding data for Gk-mRNA-M2 with each S15 homolog.

To test our model for S15 interaction we constructed a second mutation of Gk-mRNA targeting the putative GU/GC motif (Fig 5A). We hypothesized that this mutant should abolish regulation and binding of Ec-S15 and Rr-S15, and have less of an impact on the Tt-S15 and Gk-S15 interactions. The interaction between this mutant mRNA and all four S15 homologs was assessed using both our regulatory assay and *in vitro* binding assay. We find that this mutation indeed abolishes regulation of ß-galactosidase expression in response to Ec-S15 and Rr-S15, and reduces regulation in response to Tt-S15 (Fig 5B). In addition, this mutation abolishes *in vitro* interactions with each of these proteins (Fig 5C). Gk-S15 weakly binds this mutant (the dissociation constant is nearly two orders of magnitude higher than that for the native interaction), but displays significant regulatory activity. These results are consistent with our proposal that while the GU/GC motif alone is not sufficient to enable interaction between Gk-mRNA and its native binding partner, it is sufficient to allow interactions between Gk-mRNA and the other three S15 homologs. In summary, our results indicate that homologous proteins, even those that recognize the same RNA structures, do so using different structural determinants.

### S15 homologs interacting with non-homologous mRNA regulators have different conservation patterns

Our specificity data as well as existing studies indicate that the determinants for mRNA and rRNA binding are distinct [40,52]. We hypothesize that, depending on the RNA regulator present in the organism, the positions in S15 under strong selection are different. Such positions may be responsible for mRNA as opposed to rRNA recognition. To explore this hypothesis, we analyzed the *rpsO* coding sequences from sequenced microbial genomes containing each class of mRNA regulator. For the *E*. *coli*, *G*. *kaustophilus*, and *R*. *radiobacter* RNAs there are high-quality RNA alignments that provide a list of genomes containing each mRNA regulator [35–37]. For each class of RNA regulator we constructed alignments of the corresponding S15 protein coding sequences, which we will refer to by their species type (e.g. alignment of S15 sequences from organisms containing homologs of the Ec-mRNA will be referred to as the Ec-alignment). S15 is typically well-conserved and the alignments contain few if any gapped regions. The Gk-alignment was the largest at 202 sequences; the Ec-alignment had 165 sequences, and the Rr-alignment 65 sequences. In the case of the *T*. *thermophilus* mRNA regulator, no RNA alignment exists, and a cursory BLAST search did not return hits to the mRNA outside the Thermus genus. Both the Rr-S15 and Tt-S15 were omitted from further analysis due to the limited sequence alignments that could be constructed for them. In addition, this choice allows us to focus on the differences between Gk- and Ec-S15, which display very different RNA interaction behaviors based on our data. Previous mutagenesis studies for both Ec-S15 and Gk-S15 suggest they use similar, but not exactly the same, residues in recognition of their mRNA and rRNA [46,51,52] (S4 Fig).

**Fig 6:**
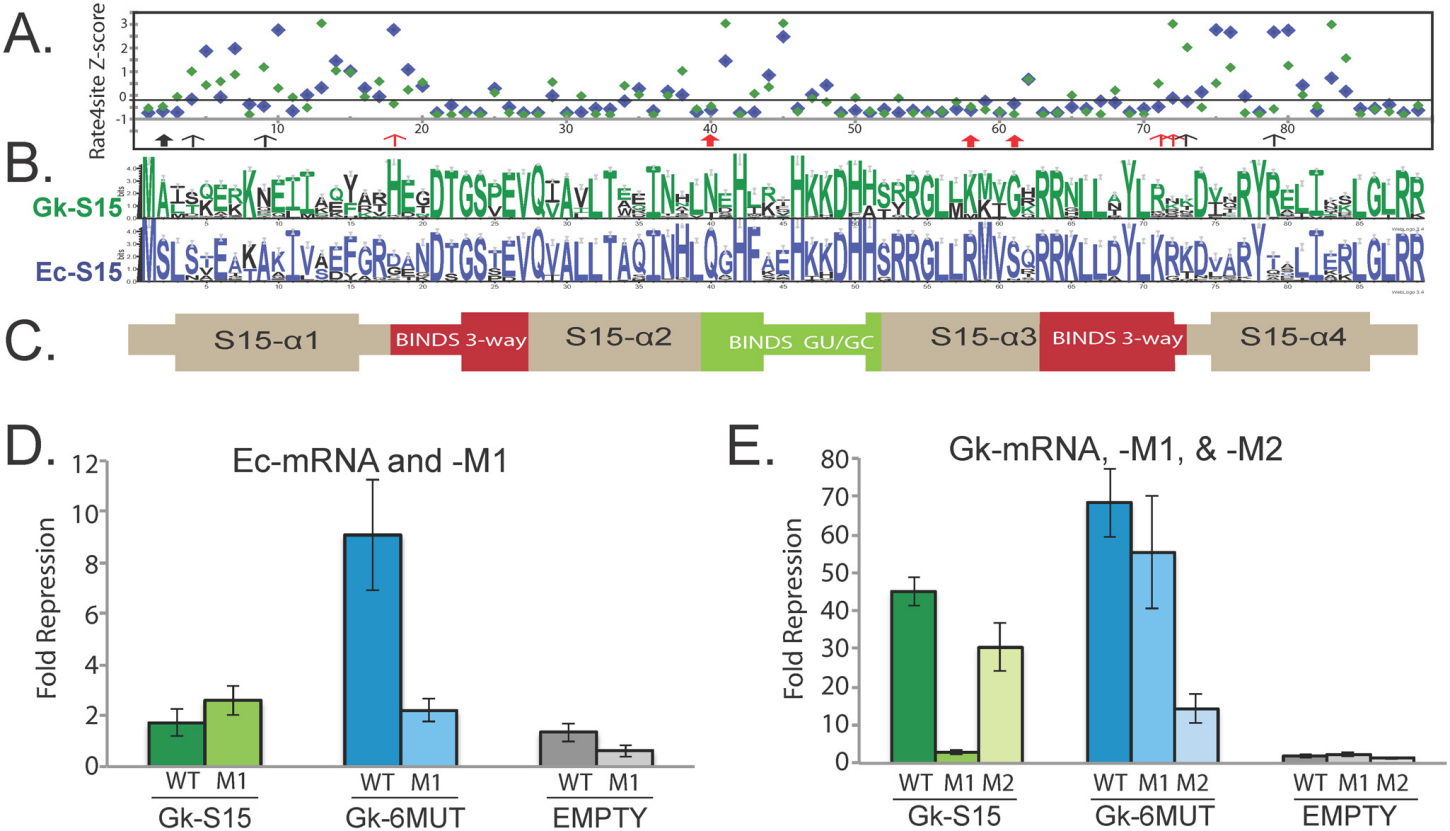
S15 protein conservation. *(A)* Rate4Site Z-value indicating degree of conservation for each alignment of S15 coding regions. Blue points correspond to values from the Ec-alignment, and green to values from the Gk-alignment. Lower values are more highly conserved positions. Solid arrows indicate positions that are conserved in both the alignments but have different amino acid identities. Open arrows indicate positions that are conserved in one alignment but not in others, red arrows indicate mutation present in Gk-S15-6MUT. *(B)* Conservation of individual amino acids within each alignment (generated with Weblogo [66]). Residue actually present in the Gk-S15 sequence colored in green, residue actually present in the Ec-S15 sequence colored in blue. *(C)* Secondary structure diagram of S15, indicating looped or alpha-helix regions, and regions that interact with either the three-way junction (red) or the GU/GC motif of rRNA (green). *(D)* Regulation of Ec-mRNA and Ec-mRNA-M1. In contrast to Gk-S15, Gk-S15-6MUT regulates Ec-mRNA, and this interaction is abolished in Ec-mRNA-M1. (E) Regulation of Gk-mRNA, Gk-mRNA-M1, and Gk-mRNA-M2. GK-S15-6MUT regulates all three of the Gk-mRNA. Error bars represent standard error for three or more replicates. Data for pEMPTY, and Gk-S15 are repeated from Fig 5 for comparison.

To systematically assess which positions might be under selective pressure, we used the tool Rate4site to evaluate each of the alignments [53]. Rate4Site returns a Z-score for each position indicating the extent of conservation. Statistical significance of the Z-score depends on the overall extent of conservation over the entire protein sequence. Therefore, due to the small size and the high degree of conservation in our alignments, no site had statistically significant Z-scores (even those that are completely conserved). However, the Z-score may be used as a rough indicator of conservation [53] (Fig 6A). There are many positions that are strongly conserved (Z-score < -0.1), however most of these have the same amino acid conserved in both alignments (e.g. position 28, which is a strongly conserved glutamine) (Fig 6B). Positions 2, 40, 58, and 61 show evidence of strong conservation of different amino acids in the two alignments (e.g. at position 2 an alanine is conserved in the Gk-alignment, but a serine in the Ec alignment). Positions 9, 18, 71, 72, 73, and 79 are strongly conserved in one alignment, but highly variable (Z-score > 0.5) in the other (e.g. position 18 is a conserved histidine in the Gk-alignment, but quite variable in the Ec-alignment). Additionally, both the N- and C-termini of the proteins show high degrees of variability in both alignments compared with the central portion that is expected to make direct contacts with the RNA.

### Mutated *G*. *kaustophilus* S15 shows altered specificity

To determine whether the positions identified above contribute to our observed interaction specificity (Fig 4), we focused on the Ec- versus Gk-alignment differences, with the goal of identifing amino acid changes that would enable Gk-S15 to recognize and regulate gene expression of its 3WJ-mutant (Gk-mRNA-M1), or Ec-mRNA, both of which had no regulatory activity with Gk-S15. Several of the positions identified are not expected to contact the RNA based on structural data (positions 2, 4, 9, and 79) [54] (Fig 6C), or are the same in the Ec-S15 and Gk-S15 sequences (position 73) (Fig 6C). Therefore we assessed whether Gk-S15 carrying the sextuple mutation to positions H18D, N40Q, K58R, G61S, R71K, and K72R (Gk-S15-6MUT) would regulate gene regulation with Ec-mRNA, or Gk-mRNA-M1 (Fig 6A).

We find that Gk-S15-6MUT is capable of regulating gene expression with both Gk-mRNA-M1 and Ec-mRNA (Fig 6D and 6E). Furthermore, this interaction appears to be specific as it is abolished in the Ec-mRNA-M1. This result suggests that one or more of the altered positions are responsible for recognition of these mRNA structures (Fig 6D and 6E). We speculate these residues contribute to higher affinity recognition of the GU/GC motif or possibly play a role in stabilizing a secondary binding site on the mRNA, independent of the GU/GC. When tested with Gk-mRNA-M2, Gk-S15-6MUT retains significant regulatory activity, evocative of that displayed by the Tt-S15 (Fig 5B). Our results suggest that Gk-S15-6MUT still recognizes the 3WJ, and the presence of either motif is sufficient to allow gene regulation (Fig 6D and 6E).

To further assess whether the diversity present in the N- and C-termini of the protein play a significant role in recognition, we also created a series of chimeric proteins for Gk-S15 and Gk-S15-6MUT where the N- and C-terminal sections were swapped from Gk-S15 to Ec-S15 (S5A–S5C Fig). From these studies we found that the N-terminal residues from Ec-S15 typically decreased the extent of regulation across the board (S5D Fig). This could be due to several factors including potential deleterious interactions between the N-terminus and other portions of the protein structure (the N-terminus represents 12 changes between Ec-S15 and Gk-S15), as well as differences in protein expression levels. The N-termini of protein coding sequences have been implicated in the past in determining expression levels[55–58]. We also found that chimeras with swapped C-terminal portions behaved very similarly, likely due to the small number of amino acid changes (three) between the two sequences. In summary, the N- and C-terminal regions of the protein are unlikely to play a large role in mRNA recognition and gene regulation, but do impact the extent of regulation observed in our regulatory assay due to alterations in effective protein concentration.

### Conclusions

The goal of this study was to assess how the differences between S15 homologs may contribute to the diversity of mRNA regulators that arise across different bacterial phyla to allow gene regulation. This work shows how the rRNA binding site for S15 may be partially mimicked in the four different mRNA regulators. We demonstrate that S15 homologs have distinct RNA binding profiles, and that even when recognizing the same RNA, different homologs are using distinct sequence features. These results suggest that either S15 has co-evolved with its mRNA regulators, or that differences between the ancestral S15 proteins lead to the development of a diverse array of RNA regulators that we observe in nature today.

## Materials and Methods

### Plasmid construction

The pRNA plasmid was constructed by modifying the reporter plasmid ptrc-Ec-mRNA-GFP from [37]. First, the ptrc promoter was replaced with the plac promoter. Complementary oligonucleotides of the lac promoter sequence flanked by the cohesive ends corresponding to a XhoI site (5’) and a EcoRI site (3’) were phosphorylated using T4 Polynucleotide Kinase, annealed, then ligated into ptrc-EcmRNA-GFP [37] digested with XhoI and EcoRI using Quick Ligase. Second, the *lacZ* gene was amplified from *E*. *coli* genomic DNA using Phusion DNA polymerase and primers containing restriction sites SalI and XbaI. The PCR product was digested and ligated into ptrc-RNA-GFP digested using the same enzymes (GFP was excised in this process). This new plasmid, pBS2-Ec-RNA, was sequence verified. Finally, the lac repressor coding sequence (*lacI* ^*Q*^ *)* was cloned into pBS2-Ec-RNA at the XhoI site. The *lacI* ^*Q*^ gene flanked by XhoI sites was amplified from *E*. *coli* genomic DNA (Strain NCM534, K12 derivative, Yale *E*. *coli* Genetic Stock Center #8256) using Taq DNA polymerase to generate pBS3-RNA. The plasmid sequence was verified by Sanger sequencing.

All mRNA sequences were cloned into the pBS3-RNA plasmid as a translational fusion with *lacZ* using primers containing EcoRI and SalI restriction sites (See S6 Fig for overview of plasmid, and S3 Table for list of primers). Translational fusions were constructed such that the first 9 amino acids originating from *E*. *coli* or *R*. *radiobacter rpsO*, 5 amino acids from *T*. *thermophilus rpsO*, or 4 amino acids from *G*. *kaustophilus rpsO*, were appended to the N-terminus the *lacZ* sequence. The *lacZ* sequence requires a start codon from the fused *rpsO* sequence. All enzymes for molecular biology were purchased from New England Biolabs unless otherwise noted. Mutations to the mRNAs were constructed by site-directed mutagenesis (S3 Table).

pS15 protein expression plasmids were constructed by amplifying the *rpsO* open reading frame from genomic DNA with a forward primer containing a SacI site plus a strong ribosome binding site that matched the *E*. *coli* ribosome binding site preceding *rpsO* and an 8 nucleotide linker (S3 Table) preceding the *rpsO* start site. The native ribosome-binding sites preceding *rpsO* from both *G*. *kaustophilus* and *T*. *thermophilus* were tested. However, these did not allow sufficient protein production to complement the *ΔrpsO* strain and were consequently abandoned. The reverse primer contained an XbaI site. After digestion, the PCR product was cloned into the pBAD33 vector (ATCC 87402) digested with the same enzymes. All pS15 were sequence verified. The Gk-S15-6MUT sextuple mutant was created using site-directed mutagenesis with primers listed on S3 Table and chimeras created by PCR assembly using pEc-S15, pGk-S15, or pGk-S15-6MUT as template DNA.

### Growth assay

K12: Δ*rpsO E*. *coli* cells were transformed with a pS15 and a single colony picked to grow cultures +/- 15 mM L-arabinose for ~16 hours in LB + 34 ug/mL chloramphenicol. Cultures were diluted to OD_600_ = 0.01 in 0.5 mL of fresh medium 24-well plates, and OD_600_ was measured for 27.5 hours. Each pS15 was performed 3+ replicates. Doubling times were calculated by taking the inverse of the slope of ln (OD_600_) in exponential phase readings.

### *LacZ* regulatory assays

K12: Δ*rpsO E*. *coli* cells (kind gift from Gloria Culver, [48]) were co-transformed with pRNA and pS15 plasmid (made competent using the Z-competent buffer system, Zymo Research). Although this strain does contain a chromosomal copy of *lacZ*, we find that it is significantly repressed by the *lacI* ^*Q*^ allele present on our reporter plasmid such that the background levels of β-galactosidase expression from the native *lacZ* are < 10–20% of those that we observe from our reporter carried on a multi-copy plasmid (S2 and S7 Figs). However, no doubt some of the experimental variation and background that we observe is due to this additional copy. For our assays, a single colony was used to start overnight cultures, grown +/- L-arabinose (15 mM) at 37°C, then diluted the next day to OD_600_ = 0.15 in fresh media (LB + 100 ug/mL ampicillin + 34 ug/mL chloramphenicol +/- 15 mM L-arabinose). At stationary phase (5 hours after dilution) 1 mM IPTG was added to induce β-galactosidase expression. After 30 minutes, 100 ug/mL spectinomycin was used to stop initiation of protein translation, and the cultures assayed immediately according to Miller [59] to determine the levels of reporter expression. Fold repression = (Miller units of–L-arabinose)/(Miller units of + L-arabinose). All RNA/S15 combinations were examined with 3+ independent replicates (typically 4). To determine the significance, all fold repression values were compared as indicated in S1 Table (data on Figs 2 and 3) and S2 Table (data on Figs 5 and 6) using a Welch’s single-tailed T-test in Microsoft Excel. Regulation was considered biologically significant if greater than 3-fold repression was observed, and the fold-repression was significantly different (p<0.05) than that observed with an empty pBAD33 vector.

### RNA preparation

DNA corresponding to the 5’-UTR of the *rpsO* gene was PCR amplified using species-specific primers with the T7-promoter sequence added within the forward primer sequence. Genomic DNA extracted from the each species was used as template. Indicated mutations were inserted to a DNA sequence using PCR primers containing the mutation. T7 RNA polymerase [60] was used to transcribe RNA and transcription reactions were purified by 6% denaturing PAGE. Bands were visualized using UV shadow, excised, and the RNA eluted (in 200 mM NaCl, 1 mM EDTA ph 8, 10 mM Tris-HCl pH 7.5) and ethanol precipitated. Purified RNA (10 pmol) was 5’-labeled with ^32^P-ATP and purified as previously described [61]. pCp labeling was performed using T4 RNA ligase with 50 pmol RNA and 50 pmol of [^32^P]-pCp. 3’-labeled RNA was isolated using Ambion MEGAclear kit.

### Protein preparation

The *rpsO* open reading frame was PCR amplified using whole genomic DNA and species-specific primers. It was cloned into pET-HT overexpression vector similarly to previously described [62]. Sequence verified plasmid was transformed into chemically competent BL-21 cells (DE3). Protein expression and purification for all four S15 homologs was conducted as described previously [37].

### Nitrocellulose filter-binding assays

A fixed amount of 5’^32^P-labeled RNA (1000 cpm, <1 nM) was renatured for 15 minutes at 42°C, then incubated with serial dilution of S15 in Buffer A (50 mM-Tris/Acetate, pH 7.5, 20 mM Mg-acetate, 270 mM KCl, 5 mM dithiothreitol, 0.02% bovine serum albumin[63]) for 30 minutes at 25°C. For RNAs originating from thermophillic organisms, assays were also conducted at 55°C, but these either did not yield a productive interaction, or the results were not significantly different from those observed at 25°C. Nitrocellulose membrane (GE Healthcare) was used to collect RNA-S15 complexes and positively charged nylon membrane (GE Healthcare) was used to collect unbound RNA under suction in a filter binding apparatus. Membranes were air-dried 5 minutes and the fraction bound quantified by imaging membranes on a phosphorimager screen. Radioactivity counts per sample on each membrane were measured using GE Healthcare STORM 820 phosphorimager and ImageQuant. For each sample the fraction bound (Fb) corresponds to the (counts nitrocellulose)/(counts nitrocellulose + counts nylon). To determine the K_D_ and the maximum fraction bound (Max%), the resulting values were fit to the equation: F_b_ = (Max%*[S15])/([S15]+K_D_) where [S15] corresponds to the concentration of S15 in the reaction. The residuals were minimized using the Solver function in Microsoft Excel to find both the Max% and the K_D_. K_D_ values given in Table 1 represent the mean of 3 or more independent binding assays ± the standard deviation.

### S15 sequence analysis

Amino acid sequences corresponding to the *rpsO* open reading frame from all bacterial species carrying each mRNA regulator were gathered based on existing RNA alignments [35–37]. These sequences were aligned using ClustalW [64], and the alignments analyzed using Rate4site [53].

## Supporting Information

S1 Fig Confirmation of *E*. *coli ΔrpsO*. *(A)* Diagram depicting genomic region of *rpsO* in *E*. *coli*, flanked by genes *pnp* and *truB*. Arrows and numbers indicate primers and primer placement. *(B) rpsO*-specific primers used with either *E*. *coli ΔrpsO* (Δ), *E*. *coli* Xl-1 (Xl1), or no template (no), then products separated using 1% agar and visualized using ethidium bromide *(C)* PCR product was generated from *ΔrpsO* strain (Δ), *E*. *coli* Xl-1 strain (Xl1), or no template (no) using the primer sets indicated *(D)* Individual colonies of the *E*. *coli ΔrpsO* strain (Δ1-Δ8) were PCR checked using primers 739+740 to confirm replacement of *rpsO* with *kanR*. *E*. *coli* strain Xl-1 (XL1) and no template (no) were amplified at the same time for size and condition controls.
(PDF)

S2 Fig The Miller Units from + L-arabinose (protein induced) and—L-arabinose (protein uninduced) conditions for each mRNA with exogenous protein expression (Ec-S15, Rr-S15, Tt-S15, Gk-S15) and empty vector (EMPTY). *(A)* Ec-mRNA and Ec-mRNA-M1, *(B)* Rr-mRNA and Rr-mRNA-M1, *(C)* Tt-mRNA and Tt-mRNA-M1, *(D)* Gk-mRNA, Gk-mRNA-M1, Gk-mRNA-M2. Solid bars are–arabinose, hatched bars are + arabinose. Dark gray bars are WT, white bars are M1, and light gray bars are Gk-mRNA-M2. Error bars represent the standard error of 3 or more independent replicates.
(PDF)

S3 Fig Nitrocellulose filter binding assays were used to measure the strength of the mRNA-S15 interaction among all homologs tested. Each curve represents three replicates. The fraction bound was calculated per individual protein concentration F_b_ = (counts nitrocellulose)/(counts total). Dots represent average ± standard error (error bars) fraction bound at each protein concentration. Solver (Microsoft Excel) was used to fit the range of variables (Protein concentration vs. F_b_) in order to find K_D_. The curve represents a line fit to each set of data points where F_b_ = (Fb_MAX_ * Protein concentration)/(Protein concentration + K_D_).
(PDF)

S4 Fig Summary of previous mutagenesis and structural data on S15 from *T*. *thermophilus*, *E*. *coli* and *Geobacillus stearothermophilus*. *(A)* Using the crystal structure of an S15-rRNA complex [67], the residues of S15 that bind rRNA are diagramed. Two distinct regions of S15 bind two highly conserved regions of rRNA for proper ribosome assembly. The three-way junction (3WJ) of rRNA binds residues in both the loop 1 and C-terminal part of alpha helix 3 (red). Residues that contact the GU/GC region of rRNA are located in the loop 2 region of S15 (green). *(B)* Ec-mRNA-binding residues [51,52]. The residues of S15 for Ec-mRNA-specific binding display some noteworthy differences from rRNA-binding. The residues involved in GU/GC recognition of rRNA are present and very important for mRNA-regulation. Additionally, the GU/GC element has been shown to be essential for Ec-mRNA auto-regulation. Therefore, it is very likely that Ec-S15 recognizes the GU/GC element of both mRNA and rRNA through residues H41, D48, and S51 (red). Ec-mRNA lacks an apparent 3WJ, instead forming a pseudoknot. The residues shown to be essential for auto-regulation are T21, G22, and Q27, so it is hypothesized that Ec-S15 recognizes and stabilizes the pseudoknot stem via these residues. Interestingly, there are many rRNA-specific binding residues that are not required for auto-regulation (yellow). The most notable of these residues, R64, Y68, and R71, are important for 3WJ-recognition in rRNA. This strongly suggests there is no direct structural equivalent to the 3WJ in Ec-mRNA, and furthermore confirms there is only topological mimicry with Ec-mRNA and rRNA in containing a second binding site. Finally, an mRNA-specific binding residue was identified, R58 (lime), which presumably binds the A bulge of the pseudoknot and is required for auto-regulation. *(C)* Gk-mRNA-binding residues [46]. The residues found to be essential for auto-regulation almost completely coincide with the residues essential for rRNA binding. These results strongly suggest both mRNA and rRNA use identical RNA-binding profiles on Gk-S15.
(PDF)

S5 Fig Chimeric Gk-Ec-S15 protein designs and results from regulatory assays. *(A)* Conservation of individual amino acids in the Firmicute phyla (Gk-S15) and the Gammaproteobacterial phyla (Ec-S15). The amino acid sequence used in all experiments for Gk-S15 is colored green, Ec-S15 colored blue (repeated from main text for clarity). *(B)* Diagram of S15, repeated from main text, indicating important rRNA-binding regions. *(C)* Design of chimeric proteins, green bars indicate the amino acid sequence matches Gk-S15, blue bars and letters indicates the amino acid sequence matches Ec-S15 for those regions of the protein. Black bars indicate the break point where amino acid sequences were swapped from one species to the other in constructing each chimera, position 18 and position 72. *(D)* Miller assay results for all chimeric proteins tested with Gk-mRNA, Gk-mRNA-M1, Gk-mRNA-M2, and Ec-mRNA.
(PDF)

S6 Fig pBS3-RNA plasmid diagram (not drawn to scale). (PDF)

S7 Fig Background β-galactosidase expression of the *E*. *coli ΔrpsO* strain assessed using Miller Assays performed under the same conditions used to assay regulator activity. (*A*) Cells that lack a pRNA reporter plasmid display ~600–1800 Miller Units. *(B)* Cells that contain a pRNA plasmid (carrying a *lacI* ^*Q*^ allele) where the *lacZ* reporter gene was replaced with a *gfp* reporter gene (pBS4) display 6–250 Miller Units. This indicates that the *LacI* ^*Q*^ carried by the high-copy pRNA plasmid significantly reduces endogenous *lacZ* expression. *(C)* Representative data from cells containing pBS3-RNA, a plasmid that contains both *LacI* ^*Q*^ repressor and *lacZ* reporter gene, shows that the *lacZ* reporter produces significant β-galactosidase activity over the endogenous levels. S2 Fig shows the β-galactosidase expression with pBS3 containing all versions of the mRNAs tested.
(PDF)

S1 Table Statistics for data on Figs 2 and 3. Interactions are considered significant if they display >3 fold-repression and have a p-value < 0.05 when compared to empty vector. For reference we have also compared the response of all mutant RNAs to both the response of the mutant with the empty vector, and the response of the unmutated RNA with in the presence of the same protein. Significant results are bolded.
(PDF)

S2 Table Statistics for data on Figs 5 and 6. Interactions are considered significant if they display >3 fold-repression and have a p-value < 0.05 when compared to empty vector. For reference we have also compared the response of all mutant RNAs to both the response of the mutant with the empty vector, and the response of the unmutated RNA with in the presence of the same protein. Significant results are bolded.
(PDF)

S3 Table Sequences and primers used for construction. Reporter Assay mRNA constructs for pBS3: Coding sequence is bolded, restriction sites in primers are underlined. Mutations to WT sequence are indicated in red.
(PDF)
