# Supplementary figures and images for: Co-evolution of Bacterial Ribosomal Protein S15 with Diverse mRNA Regulatory Structures
Source: PLoS Genet. 2015 Dec 16;11(12):e1005720. doi: 10.1371/journal.pgen.1005720 (PMC4684408; doi:10.1371/journal.pgen.1005720)

# Supplemental Figure 1

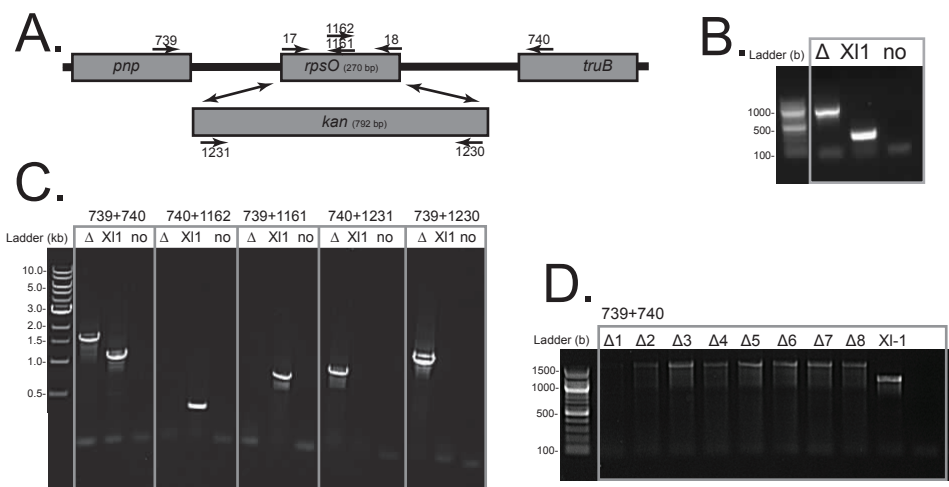

Supplement: S1 Fig — (A) Diagram depicting genomic region of rpsO in E. coli, flanked by genes pnp and truB. Arrows and numbers indicate primers and primer placement. (B) rpsO-specific primers used with either E. coli ΔrpsO (Δ), E. coli Xl-1 (Xl1), or no template (no), then products separated using 1% agar and visualized using ethidium bromide (C) PCR product was generated from ΔrpsO strain (Δ), E. coli Xl-1 strain (Xl1), or no template (no) using the primer sets indicated (D) Individual colonies of the E. coli ΔrpsO strain (Δ1-Δ8) were PCR checked using primers 739+740 to confirm replacement of rpsO with kanR. E. coli strain Xl-1 (XL1) and no template (no) were amplified at the same time for size and condition controls. (PDF) [file pgen.1005720.s001.pdf]

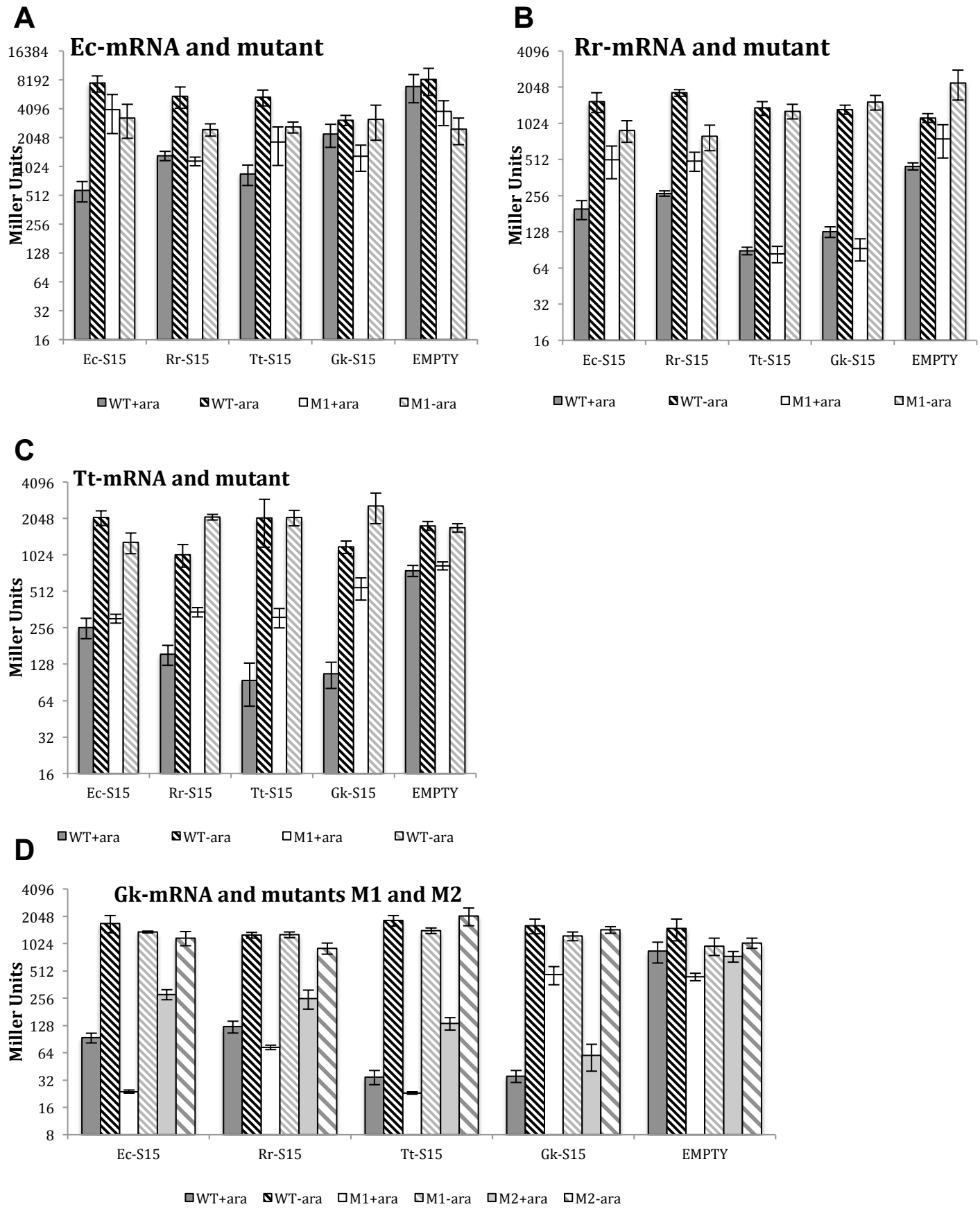

Supplementary Figure S2.

Supplement: S2 Fig — (A) Ec-mRNA and Ec-mRNA-M1, (B) Rr-mRNA and Rr-mRNA-M1, (C) Tt-mRNA and Tt-mRNA-M1, (D) Gk-mRNA, Gk-mRNA-M1, Gk-mRNA-M2. Solid bars are–arabinose, hatched bars are + arabinose. Dark gray bars are WT, white bars are M1, and light gray bars are Gk-mRNA-M2. Error bars represent the standard error of 3 or more independent replicates. (PDF) [file pgen.1005720.s002.pdf]

Supplemental Figure 3

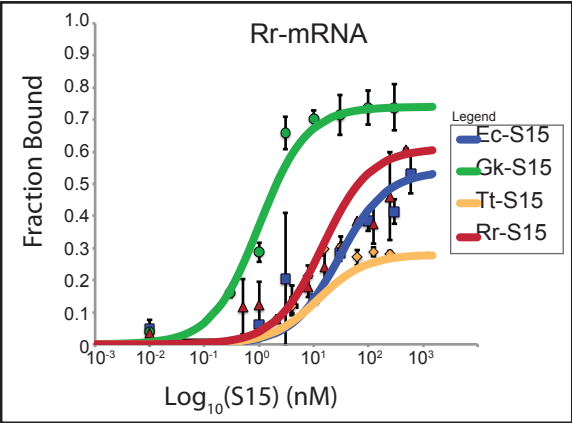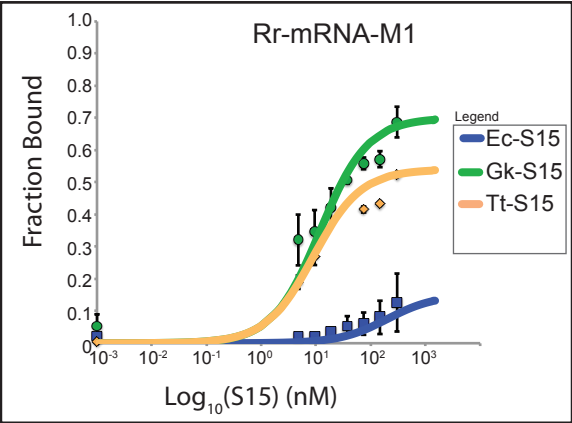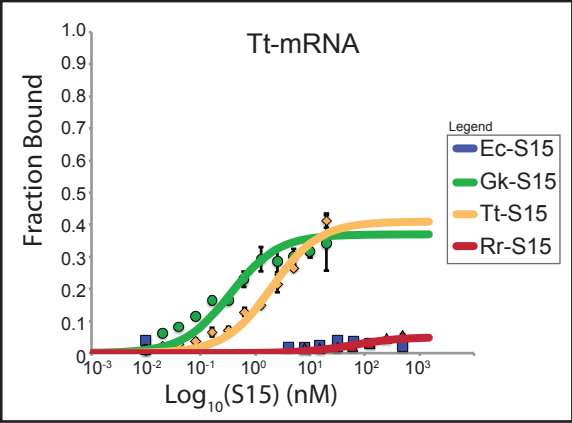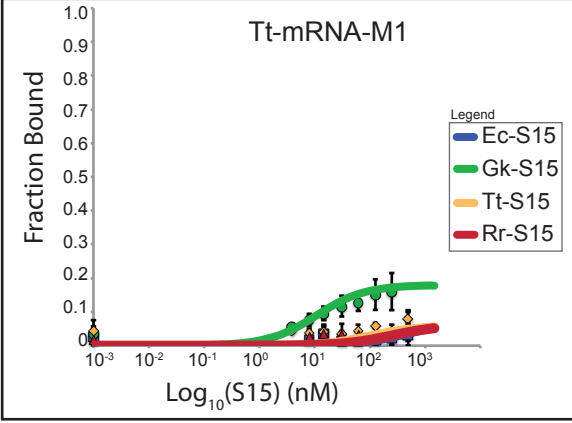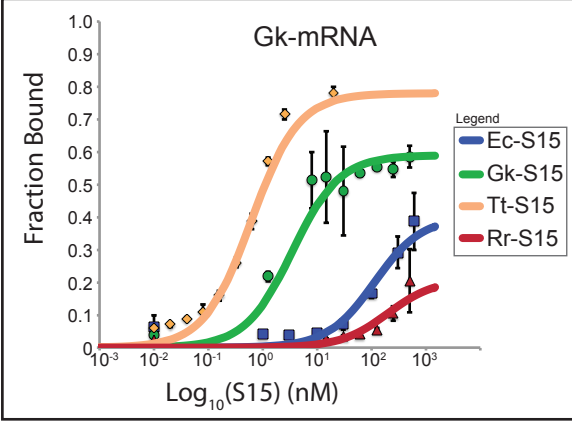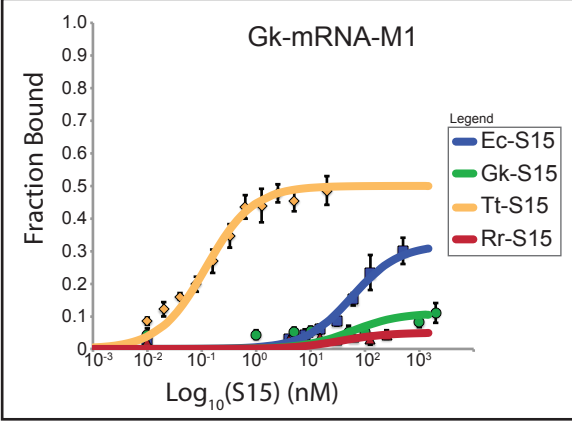

Supplement: S3 Fig — Each curve represents three replicates. The fraction bound was calculated per individual protein concentration Fb = (counts nitrocellulose)/(counts total). Dots represent average ± standard error (error bars) fraction bound at each protein concentration. Solver (Microsoft Excel) was used to fit the range of variables (Protein concentration vs. Fb) in order to find KD. The curve represents a line fit to each set of data points where Fb = (FbMAX * Protein concentration)/(Protein concentration + KD). (PDF) [file pgen.1005720.s003.pdf]

A.

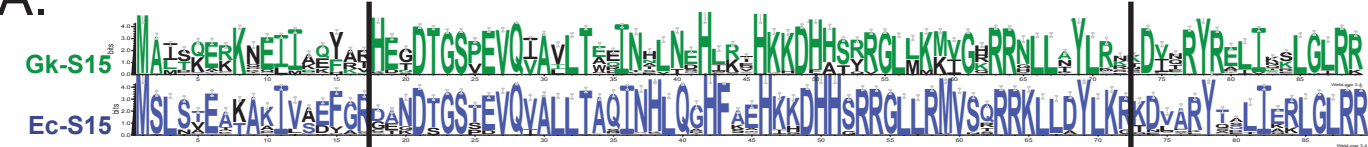

B.

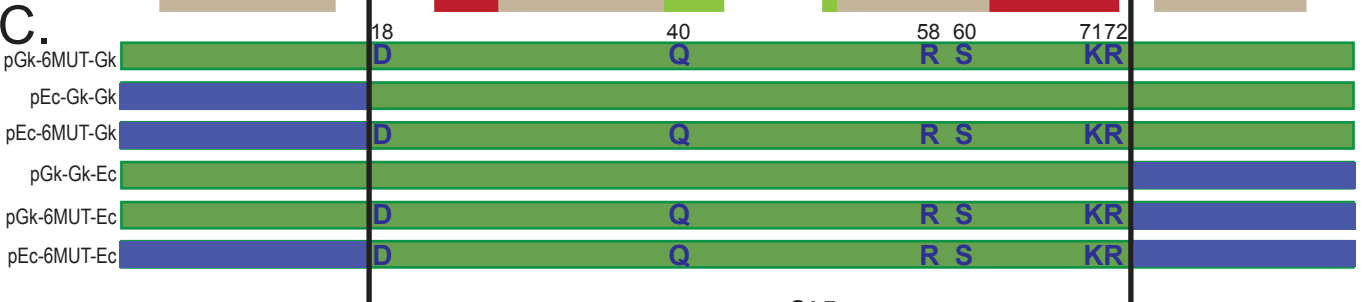

D.

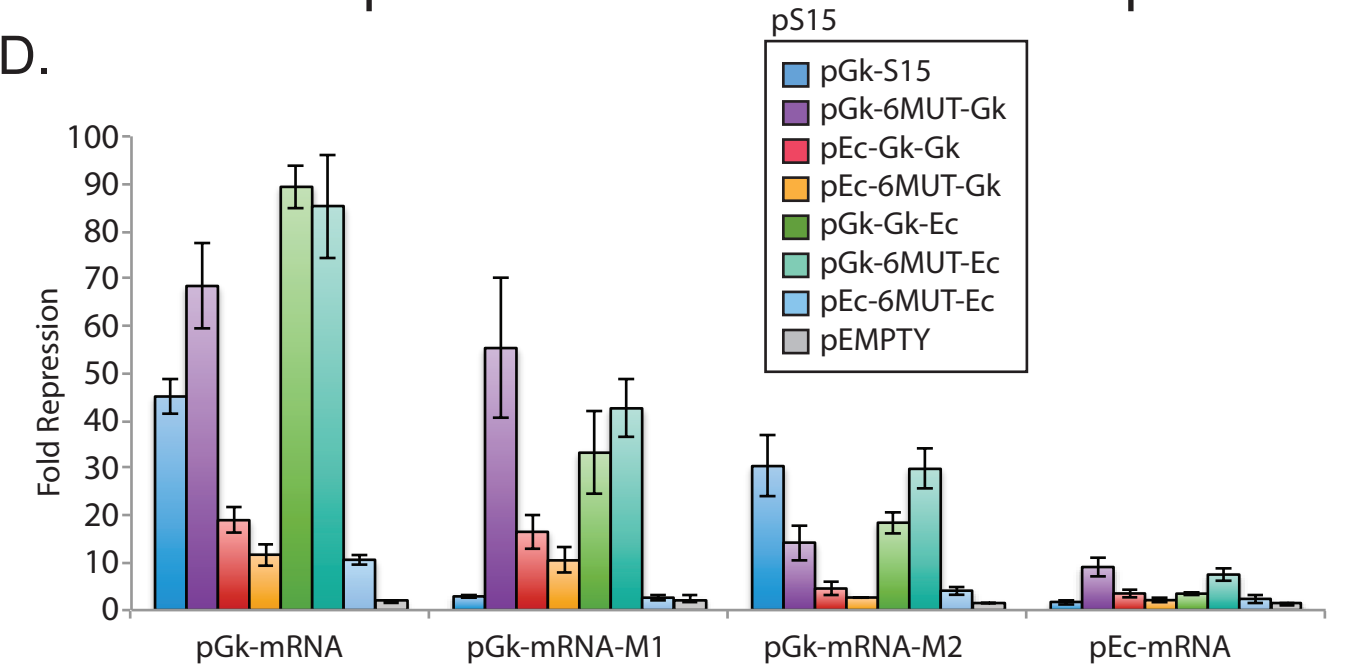

Supplement: S5 Fig — (A) Conservation of individual amino acids in the Firmicute phyla (Gk-S15) and the Gammaproteobacterial phyla (Ec-S15). The amino acid sequence used in all experiments for Gk-S15 is colored green, Ec-S15 colored blue (repeated from main text for clarity). (B) Diagram of S15, repeated from main text, indicating important rRNA-binding regions. (C) Design of chimeric proteins, green bars indicate the amino acid sequence matches Gk-S15, blue bars and letters indicates the amino acid sequence matches Ec-S15 for those regions of the protein. Black bars indicate the break point where amino acid sequences were swapped from one species to the other in constructing each chimera, position 18 and position 72. (D) Miller assay results for all chimeric proteins tested with Gk-mRNA, Gk-mRNA-M1, Gk-mRNA-M2, and Ec-mRNA. (PDF) [file pgen.1005720.s005.pdf]

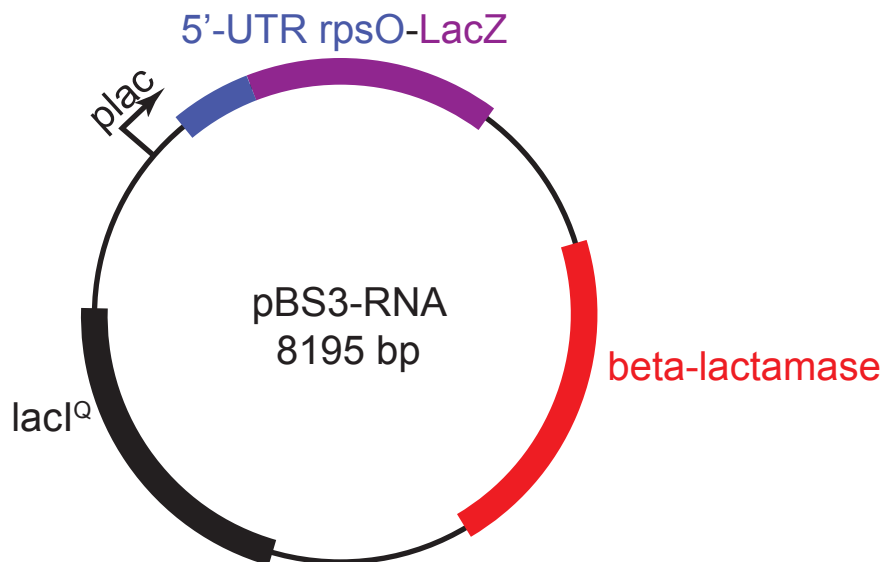

Supplemental Figure S6. pBS3-RNA plasmid diagram.

Supplement: S6 Fig — (PDF) [file pgen.1005720.s006.pdf]

Supplemental Figure 7

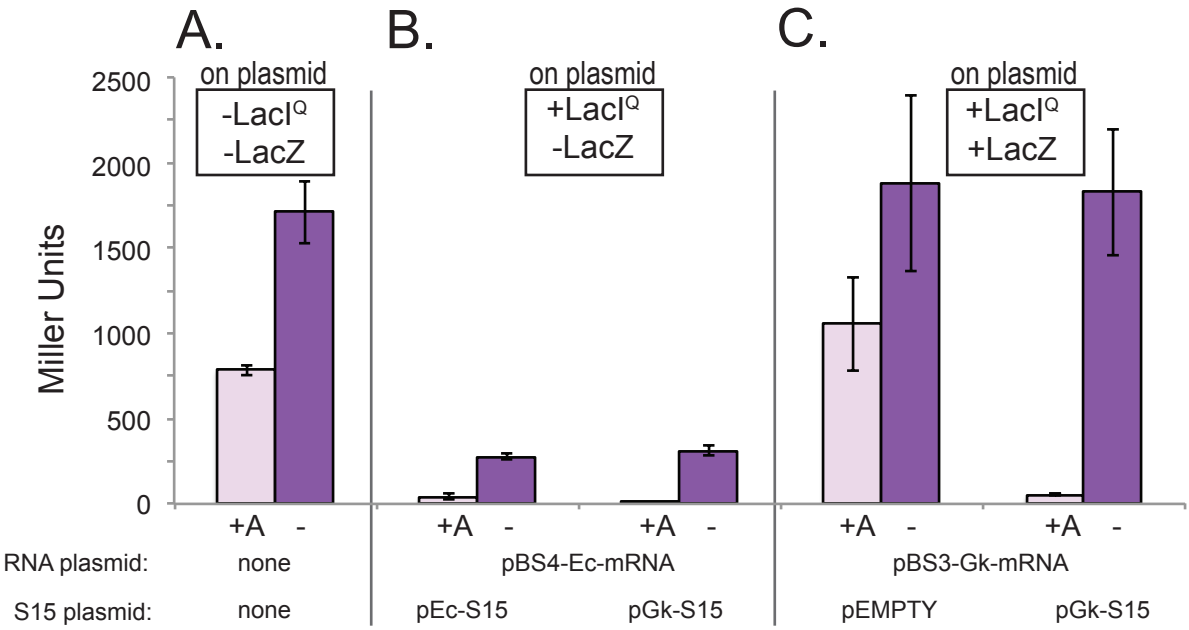

Supplement: S7 Fig — (A) Cells that lack a pRNA reporter plasmid display ~600–1800 Miller Units. (B) Cells that contain a pRNA plasmid (carrying a lacI Q allele) where the lacZ reporter gene was replaced with a gfp reporter gene (pBS4) display 6–250 Miller Units. This indicates that the LacI Q carried by the high-copy pRNA plasmid significantly reduces endogenous lacZ expression. (C) Representative data from cells containing pBS3-RNA, a plasmid that contains both LacI Q repressor and lacZ reporter gene, shows that the lacZ reporter produces significant β-galactosidase activity over the endogenous levels. S2 Fig shows the β-galactosidase expression with pBS3 containing all versions of the mRNAs tested. (PDF) [file pgen.1005720.s007.pdf]
