# Supplementary material for: Co-evolution of Bacterial Ribosomal Protein S15 with Diverse mRNA Regulatory Structures
Source: PLoS Genet. 2015 Dec 16;11(12):e1005720. doi: 10.1371/journal.pgen.1005720 (PMC4684408; doi:10.1371/journal.pgen.1005720)
Supplement: S4 Fig — (A) Using the crystal structure of an S15-rRNA complex [67], the residues of S15 that bind rRNA are diagramed. Two distinct regions of S15 bind two highly conserved regions of rRNA for proper ribosome assembly. The three-way junction (3WJ) of rRNA binds residues in both the loop 1 and C-terminal part of alpha helix 3 (red). Residues that contact the GU/GC region of rRNA are located in the loop 2 region of S15 (green). (B) Ec-mRNA-binding residues [51,52]. The residues of S15 for Ec-mRNA-specific binding display some noteworthy differences from rRNA-binding. The residues involved in GU/GC recognition of rRNA are present and very important for mRNA-regulation. Additionally, the GU/GC element has been shown to be essential for Ec-mRNA auto-regulation. Therefore, it is very likely that Ec-S15 recognizes the GU/GC element of both mRNA and rRNA through residues H41, D48, and S51 (red). Ec-mRNA lacks an apparent 3WJ, instead forming a pseudoknot. The residues shown to be essential for auto-regulation are T21, G22, and Q27, so it is hypothesized that Ec-S15 recognizes and stabilizes the pseudoknot stem via these residues. Interestingly, there are many rRNA-specific binding residues that are not required for auto-regulation (yellow). The most notable of these residues, R64, Y68, and R71, are important for 3WJ-recognition in rRNA. This strongly suggests there is no direct structural equivalent to the 3WJ in Ec-mRNA, and furthermore confirms there is only topological mimicry with Ec-mRNA and rRNA in containing a second binding site. Finally, an mRNA-specific binding residue was identified, R58 (lime), which presumably binds the A bulge of the pseudoknot and is required for auto-regulation. (C) Gk-mRNA-binding residues [46]. The residues found to be essential for auto-regulation almost completely coincide with the residues essential for rRNA binding. These results strongly suggest both mRNA and rRNA use identical RNA-binding profiles on Gk-S15. (PDF) [file pgen.1005720.s004.pdf]

Supplemental Figure 4

A.

rRNA-binding residues in S15

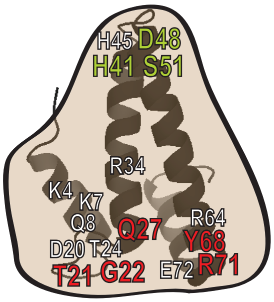

PITKEEKQKV IQEFARFPGD TGSTEVQVAL LTLRINRLSE HLKVHKKDHH SHRGLLMMVG QRRRLLRYLQ REDPERYRAL IEKLGIRG-

B.

Ec-mRNA-binding residues in Ec-S15

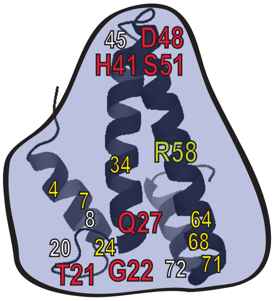

SLSTEATAKI VSEFGRDAND TGSTEVQVAL LTAQINHLQG HFAEHKKDHH SRRGLLRMVS QRRKLLDYLK RKDVARYTQL IERLGLRR-

C.

Gk-mRNA-binding residues in Gk-S15

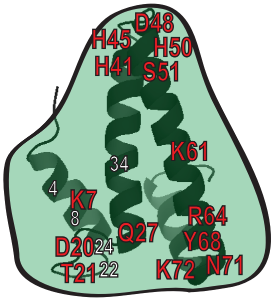

ALTQERKREI IEQFKIHEND TGSPEVQVAI LTEQINNLNE HLRIHKKDHH SRRGLLKMVG KRRNLLAYLR KKDVARYREL IEKLGLRR-
