# Supplementary material for: Co-evolution of Bacterial Ribosomal Protein S15 with Diverse mRNA Regulatory Structures
Source: PLoS Genet. 2015 Dec 16;11(12):e1005720. doi: 10.1371/journal.pgen.1005720 (PMC4684408; doi:10.1371/journal.pgen.1005720)
Supplement: S1 Table — Interactions are considered significant if they display >3 fold-repression and have a p-value < 0.05 when compared to empty vector. For reference we have also compared the response of all mutant RNAs to both the response of the mutant with the empty vector, and the response of the unmutated RNA with in the presence of the same protein. Significant results are bolded. (PDF) [file pgen.1005720.s008.pdf]

Table S1:

RNA	S15	Fold-repression (average)	St. err.	p-value	Comparison (pRNA/pS15)
**pEc-WT**	**pEc-S15**	**14.03**	**2.45**	**1.6E-03**	pEc-WT/pEMPTY
**pEc-WT**	**pRr-S15**	**3.96**	**0.51**	**1.1E-03**	pEc-WT/pEMPTY
pEc-WT	pTt-S15	0.62	1.08	5.7E-02	pEc-WT/pEMPTY
pEc-WT	pGk-S15	0.94	0.53	1.6E-01	pEc-WT/pEMPTY
pEc-WT	pEMPTY	1.36	0.37		n/a
pEc-M1	pEc-S15	1.26	0.51	1.8E-01<br>1.5E-03	pEc-M1/pEMPTY<br>pEc-WT/pEc-S15
pEc-M1	pRr-S15	2.06	0.32	1.9E-03<br>5.1E-03	pEc-M1/pEMPTY<br>pEc-Wt/pRr-S15
pEc-M1	pTt-S15	2.08	0.68	6.0E-02<br>3.4E-03	pEc-M1/pEMPTY<br>pEc-WT/pTt-S15
pEc-M1	pGk-S15	2.61	0.58	1.8E-02<br>1.5E-01	pEc-M1/pEMPTY<br>pEc-WT/pGk-S15
pEc-M1	pEMPTY	0.64	0.38		n/a
**pRr-WT**	**pEc-S15**	**8.79**	**2.89**	**5.9E-04**	pRr-WT/pEMPTY
**pRr-WT**	**pRr-S15**	**7.65**	**1.73**	**1.2E-03**	pRr-WT/pEMPTY
**pRr-WT**	**pTt-S15**	**15.43**	**1.64**	**2.09E-3**	**pRr-WT/pEMPTY**
**pRr-WT**	**pGk-S15**	**10.48**	**.71**	**1.42E-4**	**pRr-WT/pEMPTY**
pRr-WT	pEMPTY	2.53	0.22		n/a
pRr-M1	pEc-S15	2.06	0.63	1.2E-01	pRr-M1/pEMPTY
				2.6E-04	pRr-WT/pEc-S15
pRr-M1	pRr-S15	1.83	0.61	9.8E-02<br>5.6E-04	pRr-M1/pEMPTY<br>pRr-WT/pRr-S15
pRr-M1	pTt-S15	**15.54**	**0.83**	**2.7E-04**<br>4.8E-01	pRr-M1/pEMPTY<br>pRr-WT/pTt-S15
pRr-M1	pGk-S15	**17.52**	**1.55**	**3.5E-04**<br>3.7E-03	pRr-M1/pEMPTY<br>pRr-WT/pGk-S15
pRr-M1	pEMPTY	3.00	0.10		n/a
**pTt-WT**	**pEc-S15**	**8.79**	**0.76**	**2.36E-05**	pTt-WT/pEMPTY
**pTt-WT**	**pRr-S15**	**6.32**	**0.34**	**9.28E-05**	pTt-WT/pEMPTY
**pTt-WT**	**pTt-S15**	**22.69**	**3.62**	**1.47E-02**	pTt-WT/pEMPTY
pTt-WT	pGk-S15	**12.16**	**2.01**	**1.80E-02**	pTt-WT/pEMPTY

pTt-WT	pEMPTY	2.49	0.92		n/a
**pTt-M1**	**pEc-S15**	**4.38**	**0.77**	**1.5E-02**	pTt-M1/pEMPTY
				9.1E-04	pTt-WT/pEc-S15
**pTt-M1**	**pRr-S15**	**6.33**	**0.90**	**8.8E-03**	pTt-M1/pEMPTY
				5.0E-01	pTt-WT/pRr-S15
pTt-M1	**pTt-S15**	**6.85**	**0.35**	**2.3E-04**	pTt-M1/pEMPTY
				2.4E-02	pTt-WT/pTt-S15
pTt-M1	**pGk-S15**	**4.80**	**0.92**	**4.8E-02**	pTt-M1/pEMPTY
				2.5E-02	pTt-WT/pGk-S15
pTt-M1	pEMPTY	2.07	0.09		n/a
**pGk-WT**	**pEc-S15**	**18.25**	**2.95**	**5.39E-03**	pGk-WT/pEMPTY
**pGk-WT**	**pRr-S15**	**10.63**	**1.13**	**1.25E-03**	pGk-WT/pEMPTY
**pGk-WT**	**pTt-S15**	**54.05**	**4.31**	**3.20E-03**	pGk-WT/pEMPTY
**pGk-WT**	**pGk-S15**	**45.08**	**3.74**	**3.45E-03**	pGk-WT/pEMPTY
pGk-WT	pEMPTY	1.92	0.40		n/a
**pGk-M1**	**pEc-S15**	**57.84**	**3.31**	**9.1E-03**	pGk-M1/pEMPTY
				4.2E-02	pGk-WT/pEc-S15
**pGk-M1**	**pRr-S15**	**17.68**	**1.95**	**1.3E-03**	pGk-M1/pEMPTY
				1.4E-02	pGk-WT/pRr-S15
**pGk-M1**	**pTt-S15**	**61.67**	**4.38**	3.6E-04	pGk-M1/pEMPTY
				1.4E-01	pGk-WT/pTt-S15
pGk-M1	pGk-S15	2.90	0.54	2.4E-01	pGk-M1/pEMPTY
				3.4E-03	pGk-WT/pGk-S15
pGk-M1	pEMPTY	2.31	0.62		n/a
