# Supplementary material for: Co-evolution of Bacterial Ribosomal Protein S15 with Diverse mRNA Regulatory Structures
Source: PLoS Genet. 2015 Dec 16;11(12):e1005720. doi: 10.1371/journal.pgen.1005720 (PMC4684408; doi:10.1371/journal.pgen.1005720)
Supplement: S2 Table — Interactions are considered significant if they display >3 fold-repression and have a p-value < 0.05 when compared to empty vector. For reference we have also compared the response of all mutant RNAs to both the response of the mutant with the empty vector, and the response of the unmutated RNA with in the presence of the same protein. Significant results are bolded. (PDF) [file pgen.1005720.s009.pdf]

Table S2

Figure 5 Statistics

RNA	S15	av. fold	St. err.	p value	pRNA/pS15
**pGk-M2**	**pEc-S15**	**4.12**	**0.42**	4.7E-04	pGk-M2/pEMPTY
				8.1E-03	pGk-WT/pEc-S15
				8.8E-03	pGk-M1/pEc-S15
**pGk-M2**	**pRr-S15**	**4.29**	**1.03**	3.4E-02	pGk-M2/pEMPTY
				3.1E-03	pGk-WT/pRr-S15
				1.2E-03	pGk-M1/pRr-S15
**pGk-M2**	**pTt-S15**	**15.57**	**2.20**	3.8E-03	pGk-M2/pEMPTY
				1.9E-03	pGk-WT/pTt-S15
				2.1E-03	pGk-M1/pTt-S15
**pGk-M2**	**pGk-S15**	**30.40**	**6.34**	9.8E-03	pGk-M2/pEMPTY
				5.4E-02	pGk-WT/pGk-S15
				1.1E-02	pGk-M1/pGk-S15
pGk-M2	pEMPTY	1.42	0.24		

Figure 6 Statistics

**pGk-WT**	**pGk-6MUT**	**68.41**	**9.00**	3.72E-02	v pGk-WT/pGk-S15
				3.97E-03	v pGk-WT/pEc-S15
				2.54E-03	v pGk-WT1/pEMPTY
**pGk-M1**	**pGk-6MUT**	**55.31**	**14.7**	8.04E-03	v pGk-M1/pGk-S15
				4.36E-01	v pGk-M1/pEc-S15
				7.70E-03	v pGk-M1/pEMPTY
**pGk-M2**	**pGk-6MUT**	**14.24**	**3.80**	4.00E-02	v pGk-M2/pGk-S15
				1.40E-02	v pGk-M2/pEMPTY
**pEc-WT**	**pGk-6MUT**	**9.09**	**2.19**	7.24E-03	v pEc-WT/pGk-S15
				5.97E-03	v pEc-WT/pEMPTY
pEc-M1	pGk-6MUT	2.20	0.46	9.70E-03	v pEc-Wt/pGk-6MUT-Gk
				1.18E-02	v pEc-M1/pEMPTY
