# Supplementary material for: Co-evolution of Bacterial Ribosomal Protein S15 with Diverse mRNA Regulatory Structures
Source: PLoS Genet. 2015 Dec 16;11(12):e1005720. doi: 10.1371/journal.pgen.1005720 (PMC4684408; doi:10.1371/journal.pgen.1005720)
Supplement: S3 Table — Reporter Assay mRNA constructs for pBS3: Coding sequence is bolded, restriction sites in primers are underlined. Mutations to WT sequence are indicated in red. (PDF) [file pgen.1005720.s010.pdf]

Table S3:

Construct	Sequence	primers
EC m-RNA WT	ACTGGGATCGCTGAATTAGAGATCG GCGTCCTTTCATTCTATATACTTTG GAGTTTTAAA**ATGTCTCTAAGTACT GAAGCAACAGCT**	5'-GGAATTCACTGGGATCGCTGAATTAGAGATCGG
		5'-ACGCGTCGACAGCTGTTGCTTCAGTACTTAGAGACA
EC m-RNA M1	ACTGGGATTATTGAATTAGAGATCG GCGTCCTTTCATTCTATATACTTTG GAGTTTTAAA**ATGTCTCTAAGTACT GAAGCAACAGCT**	5'-TCTGGAATTCACTGGGATTATTGAATTAGAGATCGGC
		3'-same as for Ec-mRNA WT
Rr-mRNA WT	ATAGGCAGCGCCAGCTTGGGCTTTG CCTATGCTGCTGAATGGCCAGAGCT GGACGACATCCCGGCTCTAGGCGTC CCCGTTTTTCCTTAAAACAAGAAAG GATCGTACG**ATGTCGATTACTGCAG AGCGCAAAGCC**	5'-CAAGAATTCATAGGCAGCGCCAGCTTGGG
		5'-ACGCGTCGACGGCTTTGCGCTCTGCAGTAATCG
Rr-mRNA M1	ATAGGCAGCGCCAGCTTGGGCTTTG CCTATGCTGCTGAATGGCCAGAGAA AGACGACATCCCGGCTCTAGGCGTC CCCGTTTTTCCTTAAAACAAGAAAG GATCGTACG**ATGTCGATTACTGCAG AGCGCAAAGCC**	5'-GAATGGCCAGAGAAAGACGACATCCCGG
		5'-CCGGGATGTCGTCTTTCTCTGGCCATTC
Tt-mRNA WT	AGGCTTGGCGGGAGACCGCCTCGAG GAGGAAGGGGCTCAAAGCGCCTTCC CGCCTGACGGAGGGAAAAC**ATGCCC ATCACGAAG**	5'-GGAATTCAGGCTTGGCGGGAGACCGCCTCGAGGAGGAAG
		5'-ACGCGTCGACTTCGTGATGGGCATGTTTTCCCTCCGTCAGGCGGGAAG
Tt-mRNA M1	AGGCTTGGCGGGAGACCGCCAAATG GAGGAAGGGGCTCAAAGCGCCTTCC CGCCTGACGGAGGGAAAAC**ATGCCC ATCACGAAG**	5'-ACGGAATTCAGGCTTGGCGGGAGACCGCCAAATGGAGGAAG
		3'-same as for Tt-mRNA WT
Gk-mRNA WT	TCAATGTATGCGAACCATTGCTTGG CTAGGCGAGTCACCGACGCCCGCTC GGCAATCGGGGATCTAGGACTAGGG AGGTGAACAAGG**ATGGCATTGAAC**	5'-GGAATTCTCAATGTATGCGAACCATTGCTTGGC
		5'-ACGCGTCGACGTTCAATGCCATCCTTGTTCACCTCCCTAGT
Gk-mRNA M1	CGAACCATTGCTTGGCTAGGCGAGT CACCGACGCCCGCTCGGCAATCGGG GATCTAGGACTAGGGAGGTGAACAA GG**ATGGCATTGAAC**	5'-GTGGCCGAATTCCGAACCATTGCTTGGCTAGGCGA
		3'-same as for Gk-mRNA WT
Gk-mRNA M2	TCAATGTATGCGAACCATTCTCCGG CTAGGCGAGTCACCGACGCCCGCTC GGCAATCGGGGATCTAGGACTAGGG AGGTGAACAAGG**ATGGCATTGAAC**	5'-CCGGAATTCTCAATGTATGCGAACCATTCTCCGGCTAGGCGAG
		3'- same as for Gk-mRNA WT

S15 coding sequences

Sequence	Primers
pEC-S15<br>ATGTCTCTAAGTACTGAAGCAACAGCTAAAATCGTTTCT<br>GAGTTTGGTCGTGACGCAAACGACACCGGTTCTACCGAA<br>GTTCAGGTAGCACTGCTGACTGCACAGATCAACCACCTG<br>CAGGGCCACTTTGCAGAGCACAAAAAAGATCACCACAGC<br>CGTCGTGGTCTGCTGCGCATGGTTTCTCAGCGTCGTAAA<br>CTGCTCGACTACCTGAAACGTAAAGACGTAGCACGTTAC<br>ACCCAGCTCATCGAGCGCCTGGGTCTGCGTCGCTAA	5'-<br>CACGAGCTCAGGAGGTTTTAAAATGTCTC<br>TAAGTACTGAAGCACAG
	5'-<br>GCTCTAGATTAGCGACGCAGACCCAGGCG<br>C
pRr-S15<br>ATGTCGATTACTGCAGAGCGCAAAGCCGCCCTCATCACG<br>GAATATGCCACCAAGGCAGGCGACACCGGTTCTCCGGAA<br>GTTCAGGTCGCAATCCTGACCGAGCGGATCAACAACCTG<br>ACCGGTCACTTCAAGGACCACAAGAAGGACAACCACTCC<br>CGTCGTGGCCTTCTGACGCTCGTTTCGAGCCGCCGTTCG<br>CTTCTCGACTATCTGAAGAAGAAGGACGAAGCCCGTTAC<br>ACCAAGCTGATCGGTGCTCTCGGCATTCGCCGCTAA	5'-<br>CAAGAGCTCAGGAGGTTTTAAAATGTCGA<br>TTACTGCAGAGCGCAAAG
	5'-<br>CAATCTAGATTAGCGGCGAATGCCGAGAG<br>C
pTt-S15<br>ATGCCCATCACGAAGGAAGAGAAGCAGAAGGTCATCCAG<br>GAGTTCGCCCGCTTCCCCGGGGACACGGGGAGCACCGAG<br>GTGCAGGTGGCGCTCCTTACCCTGAGGATCAACCGGCTT<br>TCCGAGCACCTCAAGGTCCACAAGAAGGACCACCACTCC<br>CACCGCGGCCTCCTGATGATGGTGGGCCAGCGCCGCAGG<br>CTCCTCCGCTACCTCCAGCGGGAGGACCCCGAGCGGTAC<br>CGGGCCCTTATTGAGAAGCTGGGCATCCGGGGTTAA	5'-<br>CACGAGCTCAGGAGGTTTTAAAATGCCCA<br>TCACGAAGGAAGAG
	5'-<br>GGCTCTAGATTAACCCCGGATGCCCAGCT<br>TCTCAATAAGGGCCCG
pGk-S15<br>ATGGCATTGACGCAGGAGCGCAAACGCGAAATCATCGAG<br>CAGTTTAAAATCCATGAGAACGACACTGGTTCTCCGGAA<br>GTGCAAGTTGCGATCCTGACGGAGCAAATCAACAACTTG<br>AACGAGCATTTGCGCATTCATAAAAAAGACCATCATTCA<br>CGGCGCGGCTTGCTGAAAATGGTCGGGAAGCGCCGCAAC<br>TTATTGGCCTACTTGCGCAAGAAAGATGTGGCGCGCTAC<br>CGTGAATTGATTGAGAAACTTGGATTACGTCGATAA	5'-<br>CACGAGCTCAGGAGGTTTTAAAATGGCAT<br>TGACGCAGGAGCGC
	5'-<br>GCTCTAGATTATCGACGTAATCCAAGTTT<br>CTCAATC
pGK-6MUT<br>ATGGCATTGACGCAGGAGCGCAAACGCGAAATCATCGAG<br>CAGTTTAAAATC**G**ATGAGAACGACACTGGTTCTCCGGAA<br>GTGCAAGTTGCGATCCTGACGGAGCAAATCAACAACTTG<br>CAAGAGCATTTGCGCATTCATAAAAAAGACCATCATTCA<br>CGGCGCGGCTTGCTGCGAATGGTCAGCAAGCGCCGCAAC<br>TTATTGGCCTACTTGAAAAGGAAAGATGTGGCGCGCTAC<br>CGTGAATTGATTGAGAAACTTGGATTACGTCGATAA	GkrpsO-H18D-F 5'-<br>CATCGAGCAGTTTAAAATC**G**ATGAGAACG<br>ACACTGGTTCTCCG
	GkrpsO-H18D-R 5'-<br>CGGAGAACCAGTGTCGTTCTCAT**C**GATTT<br>TAAACTGCTCGATG
	GkrpsO-N40Q-F 5'-<br>CGGAGCAAATCAACAACTTG**CAA**GAGCAT<br>TTGCGCATTC
	GkrpsO-N40Q-R 5'-<br>GAATGCGCAAATGCTC**TTG**CAAGTTGTTG<br>ATTTGCTCCG
	GkrpsO-R71K-K72R-F 5'-<br>CAACTTATTGGCCTACTTG**AAAAGG**AAAG<br>ATGTGGCGCGC
	GkrpsO-R71K-K72R-R 5'-<br>GCGCGCCACATCTTTC**C**T**TTT**CAAGTAGG<br>CCAATAAGTTG
	GkrpsO-K58R-G60S-F 5'-<br>CGCGGCTTGCTG**CG**AATGGTC**A**G**C**AAGCG<br>CCGCAAC

Primers for *in vitro* transcription of RNAs

RNA	Primer Sequences	Template
Rr-WT	5′- TAATACGACTCACTATAGGATAGGCAGCGCCAGCTTGG	pBS3-Rr-WT
	5′- GGCTTTGCGCTCTGCAGTAATC	
Rr-M1	5′- TAATACGACTCACTATAGGATAGGCAGCGCCAGCTTGG	pBS3-Rr-M1
	5′- GGCTTTGCGCTCTGCAGTAATC	
Tt-WT	5′- TAATACGACTCACTATAGGCTTGGCGGGAGACCGCCT	pBS3-Tt-WT
	5′- TTCGTGATGGGCATGTTTTCCCTCCGTCAGGCGGGAAGGCGCTTTGAGCC	
Tt-M1	5′- TAATACGACTCACTATAGGCTTGGCGGGAGACCGCCAAATGGAGGAAG	pBS3-Tt-WT
	5′- TTCGTGATGGGCATGTTTTCCCTCCGTCAGGCGGGAAGGCGCTTTGAGCC	
Gk-WT	5′- TAATACGACTCACTATAGGTCAATGTATGCGAACCATTGC	pBS3-Gk-WT
	5′- TTCAATGCCATCCTTGTTCAC	
Gk-M1	5′- TAATACGACTCACTATAGGCGAACCATTGCTTGGCTAGGCGA	pBS3-Gk-WT
	5′- TTCAATGCCATCCTTGTTCAC	
Gk-M2	5′- TAATACGACTCACTATAGGTCAATGTATGCGAACCATTGC	pBS3-Gk-M2
	5′- TTCAATGCCATCCTTGTTCAC	

Chimeric Sequences:

Ec-Gk-Gk<br>ATGTCTCTAAGTACTGAAGCAACAGCTAAAATCGTTTCT<br>GAGTTTGGTCGTCATGAGAACGACACTGGTTCTCCGGAA<br>GTGCAAGTTGCGATCCTGACGGAGCAAATCAACAACTTG<br>AACGAGCATTTGCGCATTCATAAAAAAGACCATCATTCA<br>CGGCGCGGCTTGCTGAAAATGGTCGGGAAGCGCCGCAAC<br>TTATTGGCCTACTTGCGCAAGAAAGATGTGGCGCGCTAC<br>CGTGAATTGATTGAGAAACTTGGATTACGTCGATAA	5’-<br>GTTTCTGAGTTTGGTCGTCATGAGAA<br>CGACACTGG<br><br>5’-<br>CCAGTGTCGTTCTCATGACGACCAAA<br>CTCAGAAAC
Ec-6MUT-Gk<br>ATGTCTCTAAGTACTGAAGCAACAGCTAAAATCGTTTCT<br>GAGTTTGGTCGT**G**ATGAGAACGACACTGGTTCTCCGGAA<br>GTGCAAGTTGCGATCCTGACGGAGCAAATCAACAACTTG<br>CAAGAGCATTTGCGCATTCATAAAAAAGACCATCATTCA<br>CGGCGCGGCTTGCTGCGAATGGTCAGCAAGCGCCGCAAC<br>TTATTGGCCTACTTGAAAAGGAAAGATGTGGCGCGCTAC<br>CGTGAATTGATTGAGAAACTTGGATTACGTCGATAA	5’-<br>GTTTCTGAGTTTGGTCGT**G**ACGAGAA<br>CGACACTGG<br><br>5’-<br>CCAGTGTCGTTCTCGT**C**ACGACCAAA<br>CTCAGAAAC
Gk-Gk-Ec<br>ATGGCATTGACGCAGGAGCGCAAACGCGAAATCATCGAG<br>CAGTTTAAAATCCATGAGAACGACACTGGTTCTCCGGAA<br>GTGCAAGTTGCGATCCTGACGGAGCAAATCAACAACTTG<br>AACGAGCATTTGCGCATTCATAAAAAAGACCATCATTCA<br>CGGCGCGGCTTGCTGAAAATGGTCGGGAAGCGCCGCAAC<br>TTATTGGCCTACTTGCGCAAGAAAGACGTAGCACGTTAC<br>ACCCAGCTCATCGAGCGCCTGGGTCTGCGTCGCTAA	5’-<br>CAACTTATTGGCCTACTTGCGCAAGA<br>AAGACGTAGCACGTTACAC<br><br>5’-<br>GTGTAACGTGCTACGTCTTTCTTGCG<br>CAAGTAGGCCAATAAGTTG
Gk-6MUT-Ec<br>ATGGCATTGACGCAGGAGCGCAAACGCGAAATCATCGAG<br>CAGTTTAAAATC**G**ATGAGAACGACACTGGTTCTCCGGAA<br>GTGCAAGTTGCGATCCTGACGGAGCAAATCAACAACTTG<br>CAAGAGCATTTGCGCATTCATAAAAAAGACCATCATTCA<br>CGGCGCGGCTTGCTGCGAATGGTCAGCAAGCGCCGCAAC<br>TTATTGGCCTACTTGAAAAGGAAAGACGTAGCACGTTAC<br>ACCCAGCTCATCGAGCGCCTGGGTCTGCGTCGCTAA	5’-<br>CAACTTATTGGCCTACTTGAAAAGGA<br>AAGACGTAGCACGTTAC<br><br>5’-<br>GTAACGTGCTACGTCTTTCCTTTTCA<br>AGTAGGCCAATAAGTTG
Ec-6MUT-Ec<br>ATGTCTCTAAGTACTGAAGCAACAGCTAAAATCGTTTCT<br>GAGTTTGGTCGT**G**ATGAGAACGACACTGGTTCTCCGGAA<br>GTGCAAGTTGCGATCCTGACGGAGCAAATCAACAACTTG<br>CAAGAGCATTTGCGCATTCATAAAAAAGACCATCATTCA<br>CGGCGCGGCTTGCTGCGAATGGTCAGCAAGCGCCGCAAC<br>TTATTGGCCTACTTGAAAAGGAAAGACGTAGCACGTTAC<br>ACCCAGCTCATCGAGCGCCTGGGTCTGCGTCGCTAA	
